# Evaluation of Thermal Liquid Biopsy Analysis of Saliva and Blood Plasma Specimens as a Novel Diagnostic Modality in Head and Neck Cancer

**DOI:** 10.3390/cancers16244220

**Published:** 2024-12-18

**Authors:** Gabriela Schneider, Alagammai Kaliappan, Nathan Joos, Laura M. Dooley, Brian S. Shumway, Jonathan B. Chaires, Wolfgang Zacharias, Jeffrey M. Bumpous, Nichola C. Garbett

**Affiliations:** 1 UofL Health—Brown Cancer Center, Department of Medicine, University of Louisville, Louisville, KY 40202, USA; gabriela.schneider@louisville.edu (G.S.); alagammai.kaliappan@louisville.edu (A.K.); j.chaires@louisville.edu (J.B.C.); wolfgang.zacharias@louisville.edu (W.Z.); 2 Department of Otolaryngology Head and Neck Surgery and Communicative Disorders, School of Medicine, University of Louisville, Louisville, KY 40202, USA; nathan.joos@imail.org (N.J.); dooleyl@health.missouri.edu (L.M.D.); jeffrey.bumpous@louisville.edu (J.M.B.); 3 Department of Diagnosis and Oral Health, School of Dentistry, University of Louisville, Louisville, KY 40202, USA; brian.shumway@louisville.edu; 4 Division of Medical Oncology and Hematology, Department of Medicine, University of Louisville, Louisville, KY 40202, USA

**Keywords:** thermal liquid biopsy (TLB), differential scanning calorimetry (DSC), TLB profile, diagnosis, diagnostic modality, saliva, blood plasma, head and neck cancer (HNC)

## Abstract

Background: Over the past decade, saliva-based liquid biopsies have emerged as promising tools for the early diagnosis, prognosis, and monitoring of cancer, particularly in high-risk populations. However, challenges persist because of low concentrations and variable modifications of biomarkers linked to tumor development when compared to normal salivary components. Methods: This study explores the application of differential scanning calorimetry (DSC)-based thermal liquid biopsy (TLB) for analyzing saliva and blood plasma samples from head and neck cancer (HNC) patients. Results: Our research identified an effective saliva processing method via high-speed centrifugation and ultrafiltration, resulting in reliable TLB data. Notably, we recorded unique TLB profiles for saliva from 48 HNC patients and 21 controls, revealing distinct differences in thermal transition features that corresponded to salivary protein denaturation. These results indicated the potential of saliva TLB profiles in differentiating healthy individuals from HNC patients and identifying tumor characteristics. In contrast, TLB profiles for blood plasma samples exhibited smaller differences between HNC patients and had less utility for differentiation within HNC. Conclusions: Our findings support the feasibility of saliva-based TLB for HNC diagnostics, with further refinement in sample collection and the incorporation of additional patient variables anticipated to enhance accuracy, ultimately advancing non-invasive diagnostic strategies for HNC detection and monitoring.

## Simple Summary

In recent years, researchers have been investigating saliva as a potential tool for the early detection of cancer and to monitor patients’ health. Saliva is easy to collect, making it a good alternative to blood tests. This study evaluated a research technique called thermal liquid biopsy (TLB) to analyze saliva from patients with head and neck cancer (HNC). Our research identified an effective processing method for saliva samples, resulting in reliable TLB profiles that showed differences between healthy individuals and those with cancer. These profiles revealed important details about the types and stages of cancer present. Analyzing blood samples using TLB was less effective in identifying differences related to HNC. Our findings suggest that saliva testing using TLB could be a valuable, non-invasive way to help diagnose HNC, and with further improvements, this method could lead to better, more personalized patient care in the future.

## 1. Introduction

Early detection of cancer before it infiltrates surrounding tissues and metastasizes increases the chances of successful treatment and patient survival. As a result, substantial effort has been dedicated to identifying cancer-specific biomarkers and developing innovative diagnostic tests with high specificity and sensitivity. These methods should also be cost-effective to facilitate large-scale screening and, ideally, be non-invasive. Traditionally, most diagnostic tests have relied on blood specimens; however, recent studies have shifted focus towards saliva-based liquid biopsies for cancer diagnosis, monitoring and prognostic assessment [1]. Saliva collection is not only simple and inexpensive but presents some important advantages over blood collection in being entirely non-invasive, pain-free, and can be collected without the requirement of trained professionals [2]. Such accessibility is particularly beneficial for populations with limited healthcare resources, allowing flexible and repeated sampling to enable real-time monitoring of patients [3]. Moreover, several studies suggest that the composition of saliva can reflect changes in genomic, epigenetic, proteomic, and metabolomic profiles associated with the development of cancers located in the oral cavity, larynx and pharynx, thus indicating that a saliva-based test could play a crucial role in diagnosing head and neck cancer (HNC) [1,4,5]. Notably, HNCs account for over 4% of all cancer diagnoses in the United States and an even higher percentage worldwide [6,7]. While 5-year survival rates have improved over the past four decades from 54.6% in 1975 to 68% in 2018 [8], because of the difficulty in early diagnosis, many of these tumors present at advanced stages. Unlike many other common forms of carcinoma, there are currently no widely accepted screening modalities in place to assist with the early diagnosis of head and neck tumors. While tissue biopsy remains the most frequently used diagnostic approach for HNC, it is not without its drawbacks as this method is invasive and painful, and it often fails to capture the heterogeneity within the tumor, compromising the specificity, sensitivity and overall accuracy of the diagnosis [1]. By exploring saliva-based diagnostics, we may pave the way for earlier diagnosis and monitoring of patients with HNC.

The potential utility of saliva in liquid biopsy testing for the diagnosis and monitoring of HNC has been extensively explored. Several microsatellite markers have been identified in exfoliated oral mucosal cell samples, and more importantly, microsatellite instability has been detected in saliva samples [9]. Similarly, it has been shown that mitochondrial genes Cox I and Cox II are elevated in the saliva of patients with HNC compared to healthy controls [10]. The analysis of exosomes in saliva revealed elevated levels of CD63 [11] and several miRNAs, such as miR-486-5p, miR-486-3p, and miR10b-5p, in specimens from patients with HNC, suggesting their use as potential biomarkers [12,13]. Furthermore, several other miRNAs, such as miR-200 and miR-125a, were found to be decreased in oral cancer patients [14]. Proteomics studies identified potential biomarkers that were increased (e.g., IL-8, IL-1β, IL-6, DUSP1, HA3, OAZ1, S100P, SAT, MMP-9) [15,16,17,18,19] or decreased (e.g., annexin 1) [20], as well as some that had elevated activity (e.g., aldehyde dehydrogenase) [21] or were exclusive (e.g., α1-antitrypsin, haptoglobin) [22,23] to oral cancer.

The abundance of single biomarkers contributing only moderate diagnostic performance for HNC identification motivated us to evaluate differential scanning calorimetry (DSC), a technique that, instead of detecting a single biomarker, provides a composite profile of multiple biomarkers in complex biospecimens. DSC is an analytical technique that can be applied to characterize biological samples [24,25,26,27,28,29,30,31], where the precise detection of the heat change (excess specific heat capacity) of the sample during a controlled temperature ramp provides a specific readout of heat release (exothermic reaction) and heat absorption (endothermic reaction) associated with macromolecular transitions (e.g., protein denaturation). The result is a characteristic signature that reflects how energy is absorbed and/or released from the sample as a function of temperature. In previous work, we reported that the DSC analysis of blood plasma from healthy individuals reflects the composite denaturation transitions of highly abundant plasma proteins [29]. In contrast, the denaturation behavior of disease-state blood plasma is altered because of changes in the transitions of the abundant proteins. DSC is a highly sensitive technique that can detect heat on the microcalorie scale necessary to capture disease-related changes in the thermodynamic properties of complex biomolecule solutions [32,33]. These changes can include alterations in the levels of component biomolecules, their folding and structure, post-translational modifications, or interactions between biomolecules and disease-related metabolites. Distinct from approaches focused on the identification of the differential expression of low abundance proteins or disease metabolites, DSC provides a unique analysis of the disease proteome detecting how these low abundance components may induce structural changes, modifications, or interactions with the high abundance proteins and alter their composite denaturation behavior [29,33,34]. Over the last 15 years, a compendium of studies have reported the application of DSC for the characterization of multiple biospecimens, such as plasma, serum, or cerebrospinal fluid [24,25,26,27,28,29,30,31], with Velazquez-Campoy et al. [25] first coining the phrase thermal liquid biopsy (TLB) in 2018 to refer to the use of DSC to provide complementary diagnostic analysis of biofluids based on disease-specific changes in the thermal properties of biomolecular components. In common with other liquid biopsy techniques, the ability of DSC to provide low-cost, minimally or non-invasive, more frequent diagnostic assessment [25,35] provides advantages over some other diagnostic modalities, such as diagnostic imaging, which may be limited in frequency by factors such as cost, convenience, and radiation burden.

**Figure 1:**
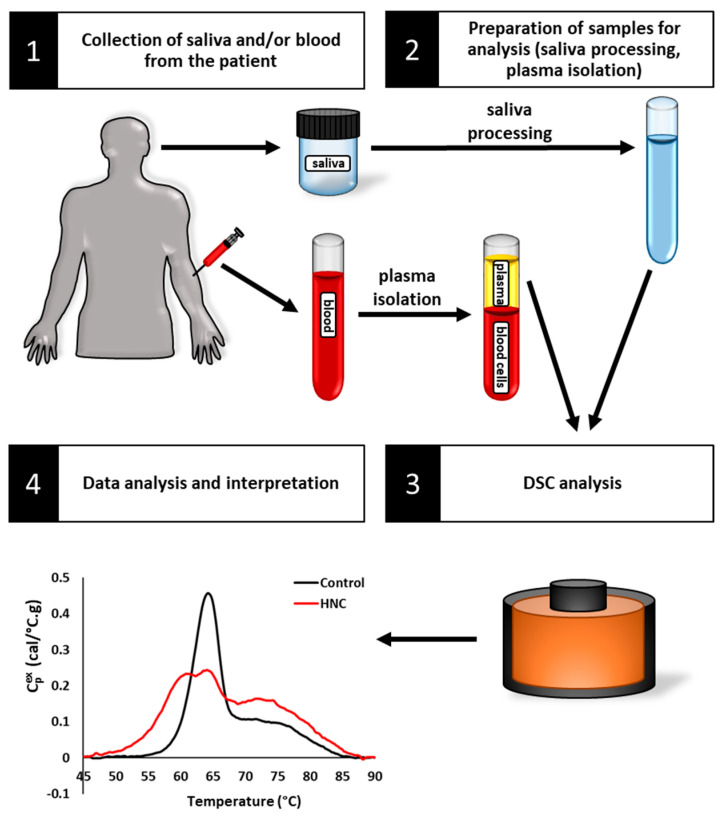
Schematic representation of the possible use of DSC-based TLB of saliva and/or plasma for screening, diagnosis, prognosis, or monitoring of HNC patients. The process begins with the collection of saliva and/or blood from patients, followed by saliva processing and/or blood plasma isolation. TLB involves DSC analysis to capture the comprehensive protein denaturation behavior of the patient saliva/plasma samples. The resulting TLB profiles are used in the detection of HNC and for further characterization of HNC for personalized monitoring of HNC patients.

In the current study, we hypothesized that the diagnostic sensitivity of thermal liquid biopsy would allow us to observe differences in TLB profiles of saliva and plasma samples obtained from HNC patients and healthy controls resulting from disease-related differences in the proteomic, transcriptomic, and metabolomic composition of saliva and plasma (Figure 1). Although the application of DSC for the analysis of blood plasma has been documented in multiple studies, its use for saliva analysis has only been reported in a single small study, where variability in TLB profiles was most likely related to sample processing parameters and DSC instrument settings [36]. In this study, our objectives were as follows: (1) to determine the feasibility of sample processing methods and TLB interpretation for salivary specimen analysis; (2) to use DSC to obtain TLB profiles for saliva samples from HNC patients and healthy controls; (3) to obtain TLB profiles for blood plasma samples from HNC patients using previously established methods; and (4) to evaluate saliva and plasma TLB profiles for trends in TLB signature as a function of tumor stage and location. To this end, we first evaluated twelve different saliva processing methods to identify the optimal method to provide reproducible saliva TLB profiles. We then compared saliva TLB profiles between patients with HNC and healthy volunteers to identify changes in saliva TLB profiles associated with HNC. Finally, we collected TLB profiles of plasma samples obtained from HNC patients to evaluate the utility of DSC to detect biofluid-specific changes related to HNC, and we evaluated saliva and plasma TLB profiles to identify which biospecimen provided the greatest diagnostic differentiation of patient status.

## 2. Materials and Methods

### 2.1. Study Design and Patient Population

This study was reviewed and approved by the Institutional Review Board at the University of Louisville (IRB# 08.0445) in compliance with the Declaration of Helsinki. Informed consent was obtained from all subjects involved in the study. De-identified saliva and plasma samples, and associated patient data, were obtained from HNC patients attending otolaryngology clinics at the University of Louisville. For each sample, information about patient diagnosis, medical history, history of social risk factors, and demographic status were provided and used for analysis (Table 1). Healthy Control saliva samples were obtained from volunteers at the University of Louisville School of Dentistry with no active cancer diagnosis. All plasma specimens were obtained by collecting blood into vacutainers containing K2 EDTA anticoagulant (Becton, Dickinson and Company, Franklin Lakes, NJ, USA), which were immediately processed, aliquoted, and stored at −80 °C until analyzed by DSC. Saliva samples were collected without any additives. Study participants were instructed to avoid food and drink for at least one hour before saliva collection. They were asked to refrain from swallowing until collecting 2–3 mL of saliva then to gently swish the saliva in their mouths for 1–2 min before spitting it into a sterile graduated specimen cup. Saliva samples were stored at −80 °C until analysis.

### 2.2. Sample Processing for DSC Analysis

Plasma samples and purified proteins were processed as described previously [33,37]. Saliva samples were thawed overnight at 4 °C before processing. To identify the optimal approach for saliva sample processing, twelve different methods were evaluated (Table 2, Figure S1). After saliva sample processing, the final supernatants/pellet suspensions were then dialyzed for 24 h at 4 °C into a standard phosphate buffer (1.65 mM KH_2_PO_4_, 8.35 mM K_2_HPO_4_, 150 mM NaCl, 14.7 mM Na_3_C_6_H_5_O_7_·2H_2_O, pH 7.5) with four buffer changes to achieve normalization of buffer conditions for all samples [25]. Samples were recovered from dialysis units and the final dialysis buffer was filtered using a 0.2 µm Supor 200 membrane disc filter, 47 mm (Pall Corporation, Ann Arbor, MI, USA). The dialysis buffer was used for sample dilution and as a reference solution for DSC analysis. Saliva samples were diluted 1.2- to 3.6-fold with dialysis buffer to obtain a suitable working concentration (0.5–2 mg/mL) for DSC analysis. To compare TLB profiles across samples, individual saliva and plasma TLB profiles were normalized in terms of the amount of total protein in each sample using a bicinchoninic acid (BCA) colorimetric assay (Pierce Biotechnology Inc., Rockford, IL, USA) in a microplate format (Tecan U.S., Research Triangle Park, NC, USA) using a Tecan Safire plate reader.

### 2.3. DSC Data Collection and Post-Processing

DSC data were collected using an N-DSC II (Calorimetry Sciences Corporation, Lindon, UT, USA), VP-Capillary DSC (MicroCal, Northampton, MA, USA), or Nano DSC Autosampler System (TA Instruments, New Castle, DE, USA). Based on expediency and instrument access, samples were analyzed on three DSC instrument platforms, all with similar capillary fixed-cell designs. We previously reported data consistency between the VP-Capillary DSC and the Nano DSC Autosampler System [25]; in this study, we additionally verified data consistency between the Nano DSC Autosampler System and the N-DSC II instrument for a subset of samples (Figure S2). DSC scans were recorded from 20 °C to 110 °C at a scan rate of 1 °C/min with a prescan thermostat of 15 min. Duplicate scans were collected for all samples. Raw DSC data were post-processed using Origin version 7 (OriginLab Corporation, Northampton, MA, USA). First, sample scans were corrected for the instrument baseline by subtracting an appropriate buffer scan. Next, scans were normalized for the gram concentration of protein. Nonzero baselines were then corrected by applying a linear baseline function. Finally, TLB profiles were plotted as excess specific heat capacity (cal/°C.g) versus temperature (°C). TLB data and clinical/demographic information for all patient samples included in this study can be found in Table S1.

### 2.4. Statistical Analysis of TLB Profiles

Analysis of TLB profiles was performed on a temperature range of 45–90 °C with an interval size of 0.1 °C through the calculation of several parameters describing the major features of the TLB profile: TLB profile width at half height (Width); total area of the TLB profile (Area); the amplitude of the transition in the temperature range 55–66 or 60–66 °C for saliva and plasma, respectively (Peak 1); the temperature of Peak 1 (T_Peak 1_); the maximum TLB profile amplitude (Max); the temperature of Max (T_Max_); and principal components (PCs) derived from the full TLB profile [38]. GraphPad Prism version 10 software was used for statistical analysis (GraphPad, La Jolla, CA, USA). Because of a limited number of samples, non-parametric tests were used. The two-group contrasts of TLB profile metrics and PCs were tested for statistical significance using the Mann–Whitney test. Differences in TLB profile metrics and PCs between four or more groups were tested for statistical significance using the Kruskal–Wallis test followed by Dunn’s multiple comparisons test. Because of the preliminary nature of this study, *p*-values obtained using the Mann–Whitney test or the Kruskal–Wallis test were not adjusted for multiple comparisons and should be considered exploratory in nature.

### 2.5. Sodium Dodecyl-Sulfate Polyacrylamide Gel Electrophoresis (SDS-PAGE)

SDS-Page separation of protein samples was performed using NuPAGE Novex Tris-Acetate Mini Gels and NuPAGE SDS Sample buffer (Invitrogen, Waltham, MA, USA) according to the manufacturer’s protocol. A total of 3–5 µg of saliva samples or purified proteins [human α-amylase (Fitzgerald, Acton, MA, USA), human serum albumin (HSA; Sigma, Burlington, MA, USA), hen egg white lysozyme (Sigma), human immunoglobulin A (IgA; Athens Research & Technology, Inc., Athens, GA, USA), human immunoglobulin G (IgG; Fluka, Buchs, Switzerland) and human immunoglobulin M (IgM; Athens Research & Technology, Inc.)] were loaded into each well. The gels were stained with 0.1% Coomassie Brilliant Blue R-250 in 40% ethanol and 10% acetic acid by heating the gel with the dye for 1 min in the microwave, followed by 15 min incubation at room temperature with gentle agitation. Next, the excess dye was removed. The gel was washed with deionized water and destained using a solution of 7.5% acetic acid and 10% ethanol via incubation at room temperature with gentle agitation until the desired background was reached. The gels were then imaged with a Gel Doc imaging system (Bio-Rad, Hercules, CA, USA).

## 3. Results

### 3.1. Selection of the Optimal Saliva Processing Method and Characterization of Saliva TLB Profiles

**Figure 2:**
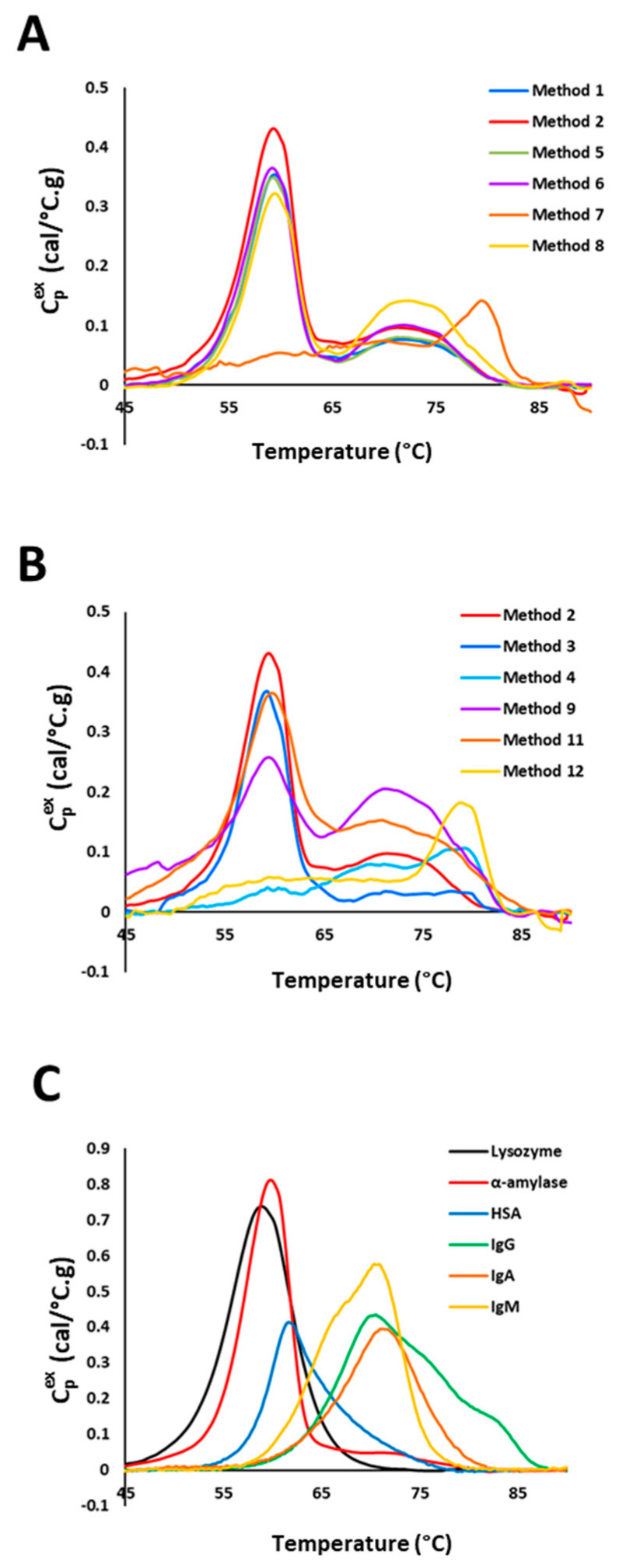
The impact of the saliva preparation method on TLB profiles. (**A**) TLB profiles of saliva samples processed using different methods incorporating centrifugation and/or filtration. (**B**) TLB profiles of saliva samples prepared using methods incorporating acid precipitation of samples. TLB profiles obtained using Method 2, identified as the most optimal processing method, is used for comparison. (**C**) TLB profiles of individual proteins abundant in saliva.

Twelve different processing methods were investigated in this study (Figure 2A,B, Figures S1 and S3) to determine reproducibility and standardization for TLB analysis, with each method performed at least twice. Methods 11 and 12 were based on the approach described by Castagnola et al. [39], where acidic treatment of saliva samples served to precipitate several salivary proteins (e.g., mucins, α-amylase, lactoferrin, immunoglobulins, carbonic anhydrase, HSA), decrease sample viscosity and inhibit intrinsic protease activity. While proteins such as HSA and immunoglobulins are major contributors to the plasma TLB profile, we decided to evaluate both the un-precipitated (Method 11) and precipitated (Method 12) fractions from this approach to evaluate how these proteins might contribute to the DSC signal for saliva TLB profiles. After sample processing, a BCA assay was used to verify the presence or absence of proteins. Samples were also analyzed using SDS-PAGE to provide a qualitative assessment of any potential impacts of the different sample processing methods (Figure S3). Since we did not detect any protein in the pellet fraction obtained using Method 10, this sample was not analyzed by DSC. Methods that included only centrifugation and/or filtration were the most optimal choices for processing because of their ability to produce consistent and defined TLB profiles. Moreover, for methods that included both centrifugation and filtration, most of the unwanted components of saliva, such as mucin, bacteria, yeast, and food particles, were removed. Results were more variable for methods using low-speed centrifugation (2700× *g*) than those using high-speed centrifugation (14,000× *g*). It is likely that lower-speed centrifugation was not as effective in the removal of unwanted saliva components as high-speed centrifugation. Processing of samples using acid precipitation (Methods 3, 4, 9–12) significantly impacted the shape of TLB profiles, particularly for the pellet fraction, with substantial differences in the position and resolution of protein denaturation transitions compared to TLB profiles resulting from other processing methods. Methods that significantly alter the solution pH from that of the physiological environment could substantially affect protein stability and are not preferred sample processing methods when a downstream application includes DSC analysis.

Also, when analyzing the samples using SDS-PAGE, by comparing to purified salivary proteins, we observed that the major proteins, such as α-amylase, lysozyme, HSA and immunoglobulins, were clearly detected in most of the samples, although there were differences in their relative amounts across processing methods. The light and heavy chains of IgG, IgM, and IgA appeared to display the most differences between samples, suggesting that the immunoglobulins are very sensitive to the sample processing method. Interestingly, when applying the chosen saliva processing method (Method 2) to patient saliva samples, bands corresponding to the light and heavy chains of these different subtypes of immunoglobulins also differed between patient samples, suggesting a wide variation in saliva composition for these proteins. Additional study regarding the potential variation in abundant salivary proteins according to the saliva processing method may be of interest, but will require more rigorous analysis, including additional controls to properly interpret the presence of various protein components and any potential protein degradation products. Ultimately, the chosen saliva processing protocol (Method 2) was selected based on the success in generating highly reproducible control TLB profiles.

Samples that were processed using centrifugation and/or filtration-based methods showed a major denaturation transition ~60 °C and a secondary transition ~70 °C. The major transition ~60 °C corresponded with the denaturation of α-amylase and lysozyme, two of saliva’s most abundant enzymatic components (Figure 2C). Another abundant saliva protein, HSA, has a denaturation transition observed ~65 °C with an asymmetric peak with a high-temperature tail that contributes to the high-temperature side of the main saliva transition ~60 °C, as well as to the secondary transition ~70 °C. Also contributing to the ~70 °C transition are the immunoglobulins, IgG (70 °C), IgA (72 °C), and IgM (71 °C). Although secretory IgA constitutes the predominant immunoglobulin isotype in saliva, all other subtypes are also present; thus, we hypothesize that the secondary saliva transition represents predominantly the composite contribution from all immunoglobulins present in saliva.

### 3.2. Comparison of Saliva TLB Profiles Between Controls and HNC Patients

A total of 48 HNC patients and 21 healthy controls were included in this study. More than 83% of patients in HNC were males, compared to 52% of the Control group. HNC patients were also slightly older with a median age of 58 years compared to 53 years for Controls. When comparing social risk factors between patient groups, as expected, nearly all HNC patients had a history of tobacco use (89.6%), and many also had a history of alcohol use (81.2%). In contrast, tobacco use (47.6%) but not alcohol use (81.0%) was lower in control subjects. Among the smokers, the average pack/year history was 50.8 for HNC patients compared to 23.5 for control subjects. For HNC patients, the most frequent tumor locations were tonsil (37.5%) and tongue (33.3%), with a lower frequency for the floor of the mouth (10.4%) and other locations (18.8%). There was an increase in the number of patients by overall cancer stage from stage I (8.3%) through stages II (20.8%) and III (31.3%), with the highest number of patients presenting with stage IV (39.6%) HNC. According to T-stage, the highest number of patients presented with T-stage II (39.6%), followed by IV (27.1%), III (20.8%), and I (12.5%). M-stage and N-stage were not included in the analysis as only one HNC patient had confirmed distant metastasis whereas changes in the American Joint Committee on Cancer staging system since data collection did not allow for a reanalysis of N-stage data. HPV and p16 viral status are presented in Table 1 for the majority of tonsil HNC patients but because of the limited sample size, these factors were not included in the analysis.

**Figure 3:**
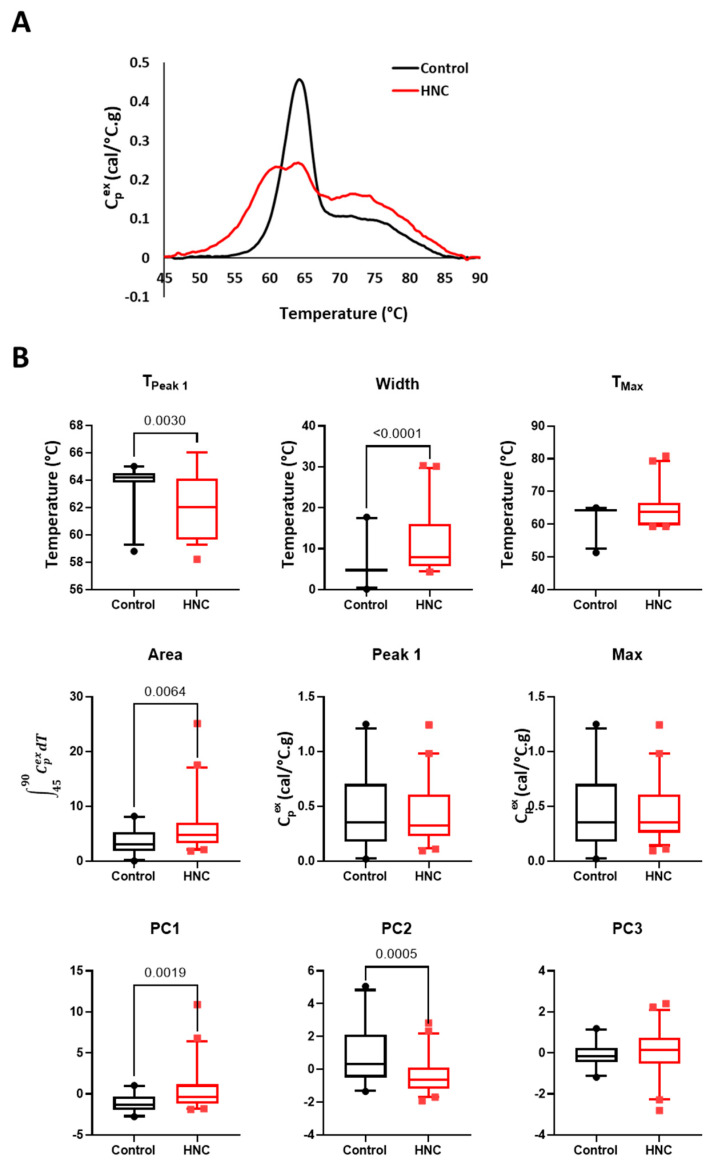
Comparison of TLB profiles of saliva samples obtained from healthy volunteers (Controls) and HNC patients. (**A**) Plot of the mean TLB profile value at each temperature for Controls (*n* = 21) and HNC patients (*n* = 48). (**B**) Boxplots of metrics and PCs calculated from TLB profiles for Controls and HNC patients. Unadjusted *p*-values < 0.05 are shown on the graphs.

A major objective of this study was to compare TLB profiles between HNC patients and Controls and to evaluate TLB changes as a function of tumor stage and location. Saliva and plasma samples were analyzed using DSC, producing a unique TLB profile for each subject. TLB profiles were sorted based on clinical group, tumor location, and tumor stage to evaluate diagnostic differences in TLB profiles. When saliva TLB profiles were evaluated based on clinical group, the mean TLB profiles for HNC patients and Controls revealed visual differences between the two groups of subjects (Figure 3A). The major denaturation transition ~60–65 °C was of lower amplitude and was broader and bifurcated for HNC patients. The bifurcated nature of the main transition was less prominent for the mean TLB profile shown by Figure 3A but more nuanced for individual TLB profiles. In general, the higher temperature maximum of the bifurcated transition was positioned at a similar temperature as the main denaturation transition for Controls, whereas the lower temperature maximum of the bifurcated transition was shifted to lower temperature. Additionally, the transition ~70–75 °C was of higher amplitude for HNC patients when compared to Controls.

To better evaluate the differences between clinical groups, we compared several metrics derived from the TLB profiles, T_Peak 1_, T_Max_, Peak 1, Max, Area, and Width. Because of the observed shift of the main transition to lower temperature for HNC saliva samples, we expanded the region of Peak 1 to 55–66 °C to better capture differences between samples. Additionally, we calculated PCs 1–3 (Figure S4), which incorporate different features of the full TLB profile into each PC. The PC loadings provide information about how strongly correlated the TLB profile value at a particular temperature is with each PC. The PC loadings plot thus provides a useful visual representation showing the regions of the TLB profile that have the largest effect on a given PC. For saliva samples, the analysis of PC loadings indicates that PC1 primarily corresponds to both main transitions, PC2 is negatively correlated with the transition ~60 °C and positively correlated with the trough located between the two main transitions, whereas PC3 is negatively correlated with the first main transition and positively with the secondary transition. As expected, based on Figure 3A, we found a significant difference in T_Peak 1_, Width, and Area (Figure 3B). We also found significant differences in PC1 and PC2 values, reflecting differences between the main transitions as well as the trough separating the transitions. Interestingly, the position of T_Peak 1_ and T_Max_ were the same for 20 out of 21 control samples, indicating that the main transition was also associated with the position of highest amplitude peak for healthy controls saliva. In contrast, for HNC saliva samples, the highest amplitude peak was positioned ~72–80 °C for 11 out of 48 samples, though the differences in the amplitude between Peak 1 and Max were minor. These results indicate a more bimodal distribution of the DSC signal for HNC samples compared to Control. Moreover, we observed more variation within the HNC group for T_Max_, Peak 1, and Max compared to Controls, which resulted in a lack of significant differences between the clinical groups.

### 3.3. Stage and Location-Dependent Differences in Saliva TLB Profiles of HNC Patients

**Figure 4:**
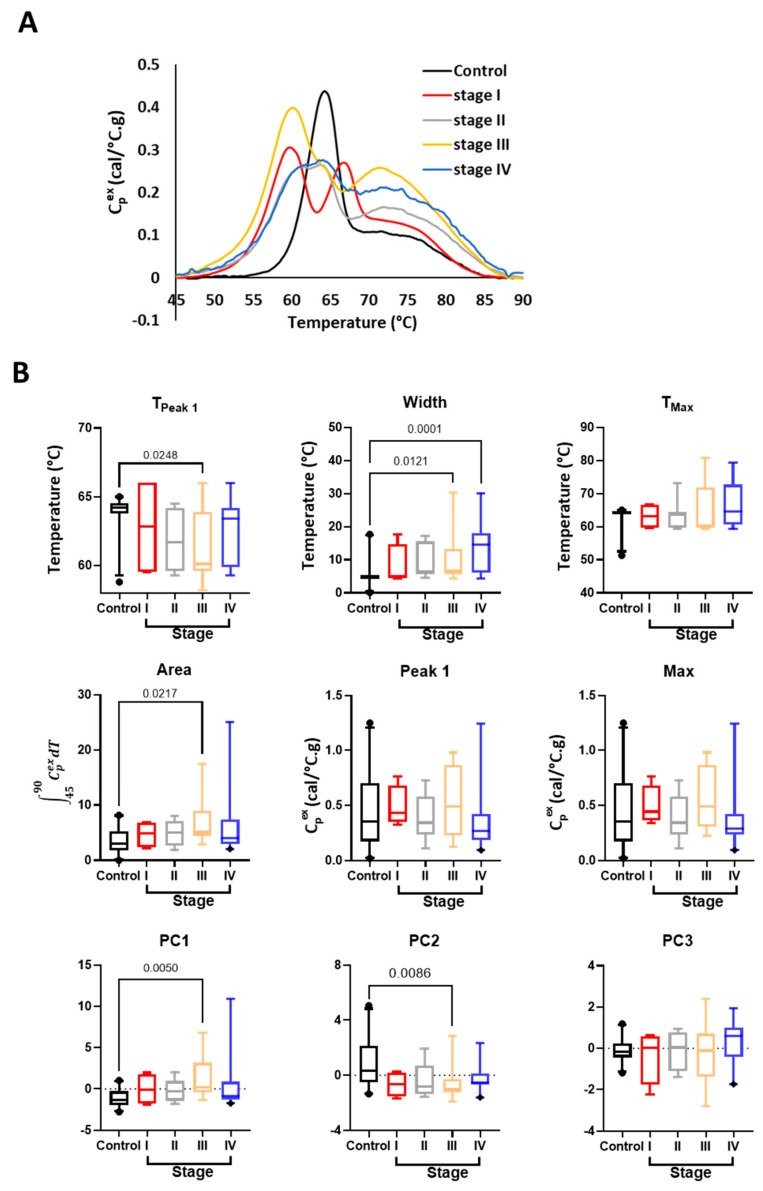
Comparison of TLB profiles of saliva samples obtained from healthy volunteers (Controls) and HNC patients separated into groups based on overall cancer stage. (**A**) Plot of the mean TLB profile value at each temperature for Controls (*n* = 21) and patients with different overall stages of HNC (Stage I, *n* = 4; II, *n* = 10; III, *n* = 15; IV, *n* = 19). (**B**) Boxplots of metrics and PCs calculated from TLB profiles for Controls and patients with different overall stages of HNC. Unadjusted *p*-values < 0.05 are shown on the graphs.

**Figure 5:**
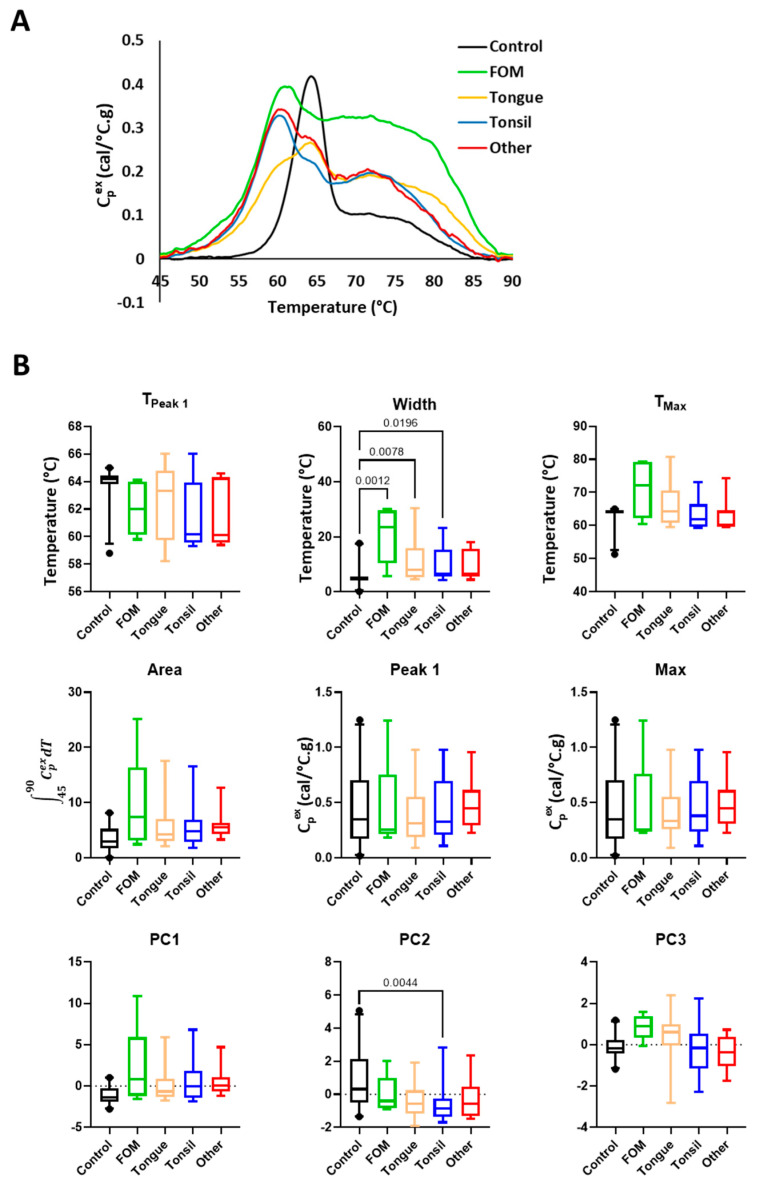
Comparison of TLB profiles of saliva samples obtained from healthy volunteers (Controls) and HNC patients separated into groups based on the cancer location. (**A**) Plot of the mean TLB profile value at each temperature for Controls (*n* = 21) and patients with different locations of HNC (FOM, *n* = 5; Tongue, *n* = 16; Tonsil, *n* = 18; Other, *n* = 9). (**B**) Boxplots of metrics and PCs calculated from TLB profiles for Controls and patients with different locations of HNC. Unadjusted *p*-values < 0.05 are shown on the graphs.

To further evaluate diagnostic changes in TLB profiles and to specifically examine whether TLB profiles are sensitive to specific cancer characteristics, TLB profiles within the HNC study group were separated by the overall stage (Figure 4 and Figure S5) or location (Figure 5 and Figure S6) of HNC. Mean TLB profiles of different overall stages of HNC revealed some visual differences between HNC stage (Figure 4A) and Controls. The most distinguishing feature between TLB profiles of Controls and overall stages of HNC was the bifurcation of the main transition ~60–68 °C, absent in Controls but present for all stages of HNC. The lower temperature maximum of the bifurcated transition ~60 °C was similar for all overall stages of HNC, whereas the position of the higher temperature maximum and degree of bifurcation were more variable. The highest degree of bifurcation was observed for Stage I HNC, with the high temperature maximum positioned ~68 °C. Stages II–IV HNC TLB profiles exhibited less-defined bifurcation and the lower temperature maximum positioned ~65 °C, with the most similar TLB profiles obtained for Stage II and IV patients. Bifurcation of the main transition was clearly visible in the mean TLB profile for the combined HNC group (Figure 3A); however, an examination of TLB profiles from individual subjects (Figure S5) revealed a high degree of variation in contrast to the more consistent TLB profiles obtained for Controls. It is perhaps not surprising that such variation in the shape of HNC TLB profiles was observed as each overall stage group consisted of different types of HNC. Despite this heterogeneity within HNC groups, we still detected significant differences for several TLB profile parameters. T_Peak 1_ values differed between Controls and Stage III HNC, which is consistent with the downshift of the major transition ~60–68 °C to ~55–60 °C. As the overall stage of HNC increased, the observation of greater temperature-shifting and complexity of protein denaturation transitions in saliva TLB profiles was captured by differences in Width between Controls and Stages III and IV HNC, as well as differences in Area between Controls and Stage III HNC. In addition, PC1, which corresponds to the two main transitions in the TLB profile, differed between Controls and Stage III HNC (Figure 4B). Similarly, PC2, which correlated with the main transition and trough located between the two main transitions, also differed between Controls and Stage III HNC. When analyzing the differences between the samples according to T-stage (associated with the size of the tumor), we observed differences in Width between Controls and T-Stages II and IV HNC, and PC1/PC2 differed between Controls and T-Stage II HNC (Figure S7).

Similarly, we observed some visual differences between TLB profiles for patients with different locations of HNC (FOM, Tongue, Tonsil, and Other). In contrast to stage, bifurcation of the main transition was most apparent in groups comprised of Tonsil and mixed HNC locations (Other), with the lower and higher temperature maxima of the bifurcated transition of similar relative amplitudes for both groups (Figure 5A). For Tonsil and FOM HNC, the main transition was positioned at ~60 °C with subtle shoulders at ~65 °C. However, for Tongue HNC, the main transition was shifted towards higher temperature of ~65 °C, like that observed for Controls, with a lower temperature shoulder ~60 °C. For the secondary TLB profile transition ~75 °C, for all HNC groups, the transition was of higher amplitude than Controls, with the FOM and Tongue groups exhibiting the broadest transition that extended beyond 85 °C. Although we did not detect statistically significant differences between groups for T_Peak 1_ and T_Max_ (Figure 5B), we observed a much wider spread of the values compared to Controls. This is not only the result of more homogenous TLB profiles observed for Controls but also reflects the large variability in TLB profiles for each of the HNC location groups (Figure S6). Interestingly, although T_Peak 1_ tends to be lower for HNC compared to Controls (reflecting the down-shift of the main transition below 60 °C), T_Max_ values, especially for FOM and Tongue samples, were higher, reflecting the shift of the highest amplitude peak in the range 72–80 °C for the majority of samples in these two subgroups. Additionally, we detected statistically significant differences in Width between Controls and all HNC patients except for mixed HNC locations (Other), which could be explained by the combination of lower sample size and high variability of this group (Figure 5B). We also observed statistically significant differences for PC2 between Controls and patients with Tonsil HNC. 

Taking into account the low number of patients, to further evaluate the effect of HNC on TLB profiles, we divided HNC samples in two main groups, namely, Tonsil (*n* = 18) and Non-tonsil (*n* = 30), where Non-tonsil consists of samples from patients with FOM, Tongue, and Other locations (Figure S8). Such grouping confirmed our previous observations that T_Peak 1_, Width, and PC1 can be used to differentiate between Controls and Tonsil HNC as well as Controls and Non-tonsil HNC, whereas changes in Area and PC2 were mainly associated with Non-tonsil HNC.

### 3.4. Stage and Location-Dependent Differences in Plasma TLB Profiles of HNC Patients

Matched saliva and plasma specimens collected from HNC patients allowed us to compare differences in TLB profiles between the two types of biospecimens. In our previous studies, we evaluated multiple parameters to encompass different features of TLB profiles that allowed us to separate clinical groups of interest [37,38,40]. In this work, we employed a similar strategy, evaluating both localized metrics as well as the PCs that incorporate multiple features of the full TLB profile into a single parameter. PCs 1–3 incorporate most of the differences between plasma TLB profiles (Figure S9), explaining more than 97% of the variance observed for the samples. PC1 positively correlates with the major transition positioned at ~63 °C and negatively correlates with the secondary transition at ~72 °C. Both PC2 and PC3 negatively correlate with the trough located between the two main transitions, whereas PC2 negatively correlates with the secondary transition and differences in the TLB profiles at ~90 °C, but PC3 exhibits the positive correlation to these regions.

**Figure 6:**
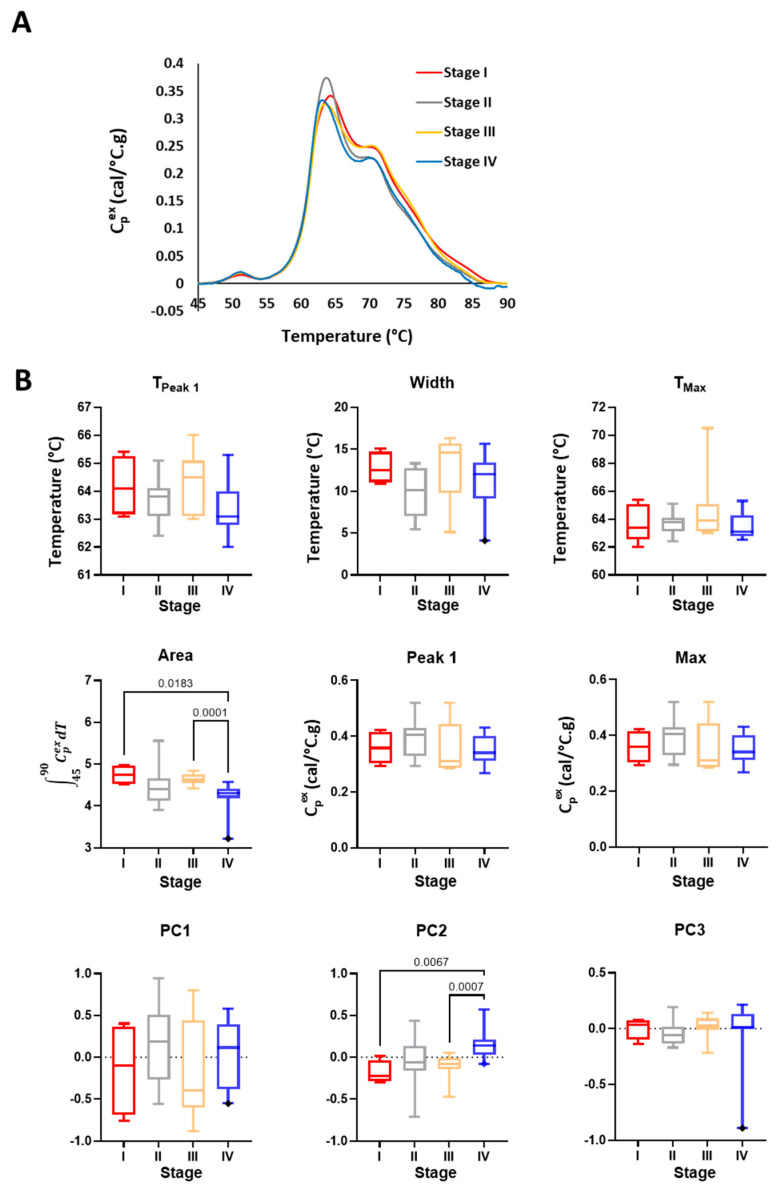
Comparison of TLB profiles of plasma samples obtained from HNC patients separated into groups based on the overall cancer stage. (**A**) Plot of the mean TLB profile value at each temperature for patients with different overall stages of HNC (Stage I, *n* = 4; II, *n* = 10; III, *n* = 15; IV, *n* = 19). (**B**) Boxplots of metrics and PCs calculated from TLB profiles for patients with different overall stages of HNC. Unadjusted *p*-values < 0.05 are shown on the graphs.

To our surprise, although we observed variation in plasma TLB profiles for individual patients within HNC overall stages (Figure S10), mean TLB profiles for each group were very similar (Figure 6A). Interestingly, PC2 values differed significantly between Stage I and Stage IV HNC patients, as well as between Stage III and IV HNC patients, and a closer visual inspection reveals an increasing trend in mean PC2 values with stage of HNC (Figure 6B). Similarly, we observed differences in Area between Stage I and Stage IV HNC patients as well as between Stage III and IV HNC; again, closer visual inspection showed a decreasing trend in Area with overall stage of cancer. PC2 values were also significantly different between T-stage I and IV HNC patients, as well as between T-stage II and IV HNC patients; and similarly, to overall stage, we also observed an increasing trend in mean PC2 values with T-stage of cancer (Figure S11).

**Figure 7:**
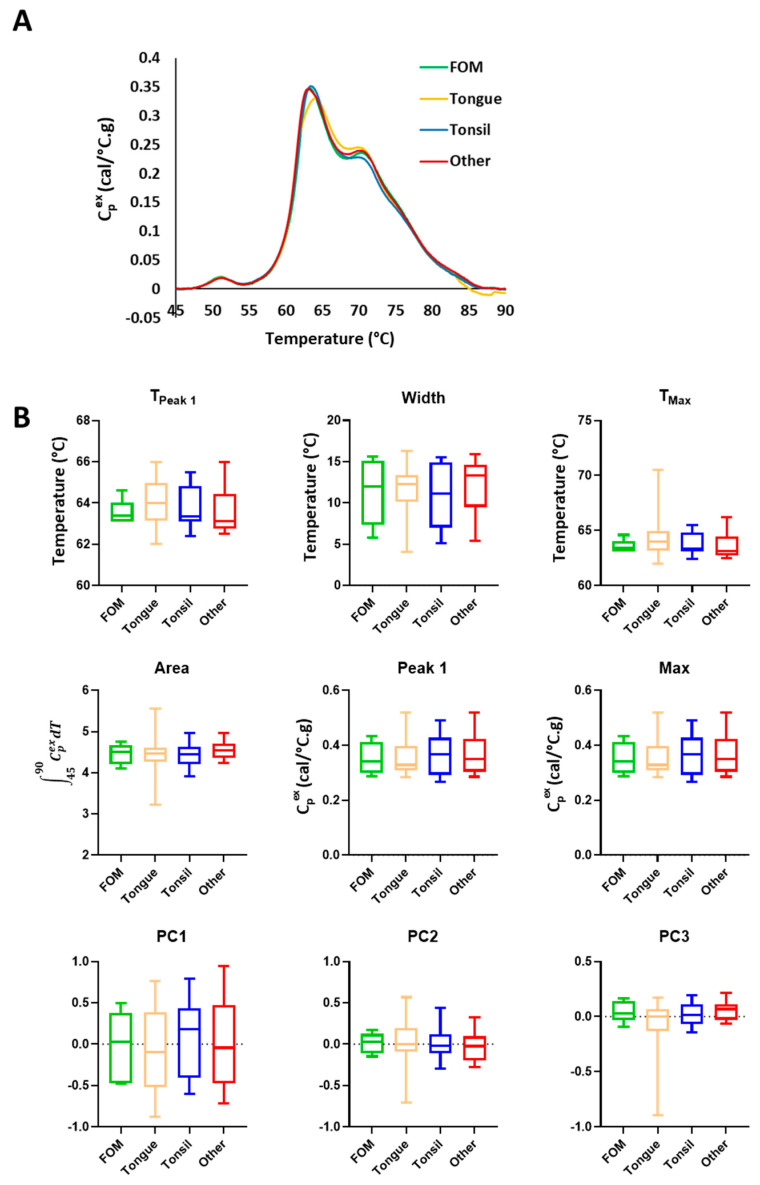
Comparison of TLB profiles of plasma samples obtained from HNC patients separated into groups based on the cancer location. (**A**) Plot of the mean TLB profile value at each temperature for patients with different locations of HNC (FOM, *n* = 5; Tongue, *n* = 16; Tonsil, *n* = 18; Other, *n* = 9). (**B**) Boxplots of metrics and PCs calculated from TLB profiles for patients with different locations of HNC. No significant differences in metrics or PCs were observed.

An evaluation of plasma TLB profiles based on cancer location (Figure 7 and Figure S12) showed the main difference was between Tongue HNC and all other sites (Figure 7A). For Tongue HNC patients, the main transition was shifted to higher temperature and the trough between the first and second transitions was not as well defined as for the other groups. However, these differences in the TLB profile features were not statistically significant when evaluating T_Peak 1,_ Width, T_Max_, Area, Peak 1, Max, and PCs 1–3 (Figure 7B), probably because of the high variability in TLB profiles within each location group that included samples with different degrees of HNC progression (Figure S12). Even when grouping patient samples into two larger groups (Tonsil vs. Non-tonsil; Figure S13), the increased sample count did not yield statistically significant differences.

## 4. Discussion

Over the last decade, a large number of studies have focused on the identification of potential cancer biomarkers in saliva, suggesting that saliva-based liquid biopsy might be used for early diagnosis, prognosis, and the monitoring of recurrence and therapy, as well as a screening tool for high-risk populations [1,4,14,19]. The major obstacle for saliva-based liquid biopsy remains the relatively low and fluctuating concentration or modification of molecules affected by tumor development against the background of normal counterparts [1]. Moreover, to increase the sensitivity and specificity of diagnostic tests, combining the levels/modifications of several biomarkers might provide more information about the presence or characteristics of cancer than single biomolecules. To address these hurdles, new approaches allowing for more comprehensive saliva analysis have been evaluated. Surface-enhanced Raman spectroscopy (SERS) successfully differentiated between the saliva of healthy individuals and oral cancer patients. This was achieved using principal component analysis based on several SERS bands assigned mainly to amino acids and proteins [41]. Another approach that captures the full biomolecular makeup of the sample is DSC, which our research group and others are evaluating for potential application in multiple clinical settings [27,30,31,35,37,42,43].

For the last ~15 years, several groups have successfully used a DSC-based thermal liquid biopsy (TLB) approach to differentiate healthy subjects from patients with a variety of cancers (i.e., cervical, breast, colorectal, brain). Studies have mainly focused on blood plasma and serum, although encouraging preliminary results have also been obtained for cerebrospinal fluid (CSF) and digested cancer tissue [24,30]. Only one preliminary study has examined DSC analysis of saliva, even though this biofluid has some important advantages over blood or CSF [36]. Saliva collection is non-invasive and does not require the involvement of health care professionals. Moreover, it is easy to perform, even for children, the elderly, and patients where the collection of blood samples might be difficult [2,3]. Therefore, in the current work, we applied DSC-based TLB for saliva sample analysis in the HNC setting. We first evaluated the most reliable saliva processing methods for DSC analysis before determining the diagnostic utility of TLB of the local cancer environment (saliva samples) compared with the peripheral environment (blood plasma) in HNC patients. TLB has gained recognition as a potentially useful and novel diagnostic technology that profiles systemic changes in the circulating proteome in the disease-state that affect conformations, modifications, interactions, or concentrations of proteins. Although DSC does not identify protein-specific changes, it provides highly sensitive detection of proteomic changes as a whole and can be used to create a TLB diagnostic signature. Thus, TLB profiles for healthy controls can be contrasted against those for individuals with known disease states, as well as contrasting within disease states. Companion studies can then identify and relate protein changes to TLB signatures capturing a diagnostic readout of the disease process. While changes in salivary protein expression are seen in HNC, potential effects on protein interactions, conformations or modifications within the saliva proteome, along with their associated TLB signatures, have yet to be defined.

To establish the use of TLB for saliva samples, the first major objective of our study was to determine the feasibility of saliva sample processing methods for TLB analysis of saliva specimens. We evaluated several sample processing techniques to obtain reliable DSC data and demonstrated that high-speed centrifugation and ultrafiltration of saliva samples through 0.45 µm filters was successful in producing reproducible TLB profiles. These observations align with the first published study of the DSC analysis of saliva samples by Pultrone et al. [36], which similarly demonstrated that centrifugation and ultrafiltration seemed to provide the most optimal processing method for saliva samples when analyzed by DSC.

Having established a reliable method of saliva processing, we successfully obtained unique TLB profiles for saliva samples from 48 HNC patients and 21 Controls. Using our established protocol for blood plasma samples, we also obtained unique TLB profiles for paired plasma samples from all 48 HNC patients. TLB profiles were sorted by clinical group, tumor location, and tumor stage to evaluate trends in TLB profiles. We demonstrated that TLB profiles of healthy volunteers’ saliva mirrored the two expected denaturation transitions of abundant salivary proteins at ~65 °C (α-amylase and lysozyme) and ~75 °C (immunoglobulins IgG, IgA, and IgM), with the denaturation of HSA contributing to both of these transitions. The position of the second transition is consistent with Pultrone and collaborators [36]; however, these authors observed the first transition at a lower temperature, between 40 and 50 °C. This may have occurred because of differences in saliva collection technique and calorimeters. First, our study collected unstimulated saliva, whereas the Pultrone study [36] collected both unstimulated and stimulated saliva samples. However, because the two collection methods did not differ statistically in protein concentrations, the authors only provided results for the stimulated saliva profiles and did not report whether the collection method had any impact on TLB profiles. Second, different calorimeters with significantly different scanning rates (1 °C/min in the current study vs. 1 °C/h in the Pultrone study [36]) were employed in these studies. The authors noted that the slow scanning rate could negatively impact the stability of the proteins present in the samples with possible protein degradation during the longer analysis time that could affect protein thermal stability (shifting protein denaturation transitions to lower temperatures). The authors also commented on the rather high variability in TLB profiles because of the daily variations in the saliva composition and amount; in addition, variation between repeated measures of the same sample was evidenced from their presented results. Our study did not evaluate the impact of daily variation in saliva composition within a subject [44] but did assess measurement reproducibility. We did not encounter issues with significant differences in TLB profiles between duplicates, lending support to the authors’ hypothesis that the slow scanning rate of the DSC instrument employed in their study negatively impacts measurement reproducibility and suggests that faster scan rates are recommended in future investigations.

Having demonstrated a generally consistent TLB profile for control saliva samples, we evaluated the diagnostic utility of both saliva and plasma TLB profiles in HNC. To quantitatively compare TLB profiles, we selected several parameters corresponding to the major features of the protein denaturation transitions. TLB profiles of saliva obtained from HNC patients showed major differences in T_Peak 1_, Width, Area, PC1, and PC2 when compared to healthy volunteers. When analyzing differences in saliva TLB profiles within HNC, we observed a large TLB profile variation for overall cancer stage and location. However, we still detected significant parameter differences between clinical groups. For example, Width differed between Controls and Stage III and IV HNC, and T_Peak 1_, Area, PC1, and PC2 displayed significant differences between Controls and Stage III. Width was also useful in the differentiation of Controls and HNC grouped by location. Interestingly, the Tonsil HNC group was significantly different from Controls when analyzing Width and PC2. Given the significant heterogeneity among patients, such as differences in age, sex, smoking status, and cancer characteristics, as well as the absence of standardized saliva collection times and conditions, which have all been shown to affect saliva composition [44,45,46], we hypothesize that optimizing sample collection and incorporating patient characteristics as variables in our analysis could further improve the accuracy of saliva-based TLB. In contrast, although some differences were observed between TLB profiles of plasma from HNC patients, the differences between individual samples were not as significant as those observed for saliva. This suggests that saliva TLB profiles might carry more information about the clinical status of HNC patients and that a larger study is warranted to better evaluate the effect of different clinical factors on saliva TLB profiles.

Our study had a number of important limitations. First, the study was limited by its sample size. The intent of this work was to report results from an initial exploratory study to assess the feasibility of saliva sample processing for TLB analysis and to determine the potential utility of TLB in the HNC setting. We were successful in identifying a saliva sample processing method that resulted in reproducible control TLB profiles and observed some important differences in TLB profiles between HNC patients and control subjects. However, the variability between individual samples when grouping by tumor location or stage could not be delineated based on the limited sample size. A larger sample size would allow for more in-depth analysis of TLB profiles related to clinical factors associated with HNC. Second, although we used several TLB profile parameters to provide a quantitative analysis of differences between TLB profiles, we were not able to investigate more detailed models to evaluate and extrapolate potential diagnostic differences. An expanded study would allow for the application of additional statistical and machine learning approaches that could improve diagnostic performance. Third, limited sample size or the effects of TLB profile outliers/variability may be obscuring the overall interpretation of mean TLB profiles in this study and thus provides important challenges to advancing the clinical application of this approach. The limitations of this study and future application of TLB in the HNC setting will be the topics of future work to refine the method in an appropriately designed and powered follow-up study with expanded cohorts selected for further stratification of confounding factors. The findings from this study warrant further investigation in larger cohorts given the potential for TLB to provide a complementary diagnostic approach for HNC.

## 5. Conclusions

In conclusion, this work demonstrated the potential of DSC-based saliva TLB for the diagnosis and monitoring of HNC. Saliva TLB profiles not only differ between healthy control and HNC groups but may also facilitate the identification of tumor location and its stage. With further optimization in sample collection and the incorporation of patient variables, such as age, sex, and social risk factors, into the analysis, we hope to refine the accuracy and reliability of this diagnostic approach, ultimately paving the way for the development of a standardized, non-invasive test for early detection and personalized monitoring of HNC patients.

## Figures and Tables

**Table 1:** Characteristics of the patients in the study cohort.

	HNC Patients (*n* = 48)	Controls (*n* = 21)
**Age, median (range)**	58 (38–79)	53 (33–65)
**Sex, *n* (%)**		
Female	8 (16.7%)	10 (47.6%)
Male	40 (83.3%)	11 (52.4%)
**Ethnicity, *n* (%)**		
White	40 (83.3%)	19 (90.5%)
Black	8 (16.7%)	2 (9.5%)
**Smoking history, *n* (%)**		
Current	28 (58.3%)	2 (9.5%)
Former	15 (31.3%)	8 (38.1%)
Never	5 (10.4%)	11 (52.4%)
**Alcohol history, *n* (%)**		
Current	28 (58.3%)	16 (76.2%)
Former	11 (22.9%)	1 (4.8%)
Never	7 (4.2%)	4 (19.0%)
Unknown	6 (14.6%)	0 (0%)
**Cancer location, *n* (%)**		
FOM	5 (10.4%)	NA
Tongue	16 (33.3%)	
Tonsil	18 (37.5%)	
Other	9 (18.8%)	
**Cancer overall stage, *n* (%)**		
I	4 (8.3%)	NA
II	10 (20.8%)	
III	15 (31.3%)	
IV	19 (39.6%)	
**Cancer T-stage, *n* (%)**		
I	6 (12.5%)	NA
II	19 (39.6%)	
III	10 (20.8%)	
IV	13 (27.1%)	
**HPV status, *n* (%)**		
Positive	15 (31.3%)	0 (0%)
Negative	4 (8.3%)	0 (0%)
Not reported	19 (39.6%)	21 (100%)
**p16 status, *n* (%)**		
Positive	8 (16.7%)	0 (0%)
Negative	11 (22.9%)	0 (0%)
Not reported	19 (39.6%)	21 (100%)

Legend: FOM, floor of the mouth; HNC, head and neck cancer; NA, not applicable; HPV, human papillomavirus. Cancer location: “Other” includes buccal mucosa, hypopharynx, oropharynx, oropharynx with tonsil, pharyngeal wall, soft palate, vallecular.

**Table 2:** Summary of different saliva processing methods.

Method	Handling Steps
**1.**	Centrifuge at 14,400× *g*, 10 min, 4 °C, Beckman Coulter Microfuge 18 Microcentrifuge (Beckman Coulter, Inc., Fullerton, CA, USA) Isolate supernatant
**2.**	Centrifuge at 14,400× *g*, 10 min, 4 °C, Beckman Coulter Microfuge 18 Microcentrifuge Isolate supernatant Filter through 0.45 µm Supor 32 mm syringe filter (Pall Corporation, Port Washington, NY, USA)
**3.**	Centrifuge at 14,400× *g*, 10 min, 4 °C, Beckman Coulter Microfuge 18 Microcentrifuge Isolate supernatant Add phosphate buffer pH 2.5 to supernatant (1:1 ratio) Centrifuge at 8300× *g*, 10 min, 4 °C, Beckman Coulter Microfuge 18 Microcentrifuge Isolate supernatant and pellet (pellet used for Method 4)
**4.**	Use pellet from Method 3, step e Wash twice with phosphate buffer, pH 2.5 Suspend in phosphate buffer, pH 7.5 (approx. 1.6× the starting volume)
**5.**	Centrifuge at 14,400× *g*, 10 min, 4 °C, Beckman Coulter Microfuge 18 Microcentrifuge Isolate supernatant Filter through 0.8 µm Supor 25 mm syringe filter
**6.**	Filter through 0.45 µm Supor 32 mm syringe filter
**7.**	Centrifuge at 2700× *g*, 15 min, 4 °C, Beckman Coulter Microfuge 18 Microcentrifuge Isolate supernatant
**8.**	Centrifuge at 2700× *g*, 15 min, 4 °C, Beckman Coulter Microfuge 18 Microcentrifuge Isolate supernatant Filter through 0.45 µm Supor 32 mm syringe filter
**9.**	Centrifuge at 2700× *g*, 15 min, 4 °C, Beckman Coulter Microfuge 18 Microcentrifuge Isolate supernatant Add phosphate buffer pH 2.5 to supernatant (1:1 ratio) Centrifuge at 8300× *g*, 10 min, 4 °C, Beckman Coulter Microfuge 18 Microcentrifuge Isolate supernatant and pellet (pellet used for Method 10)
**10.**	Use pellet from Method 9, step e Wash twice with phosphate buffer, pH 2.5 Suspend in phosphate buffer, pH 7.5 (approx. 1.6× the starting volume)
**11.**	Centrifuge at 2700× *g*, 10 s, 4 °C, Beckman Coulter Microfuge 18 Microcentrifuge Isolate supernatant Add phosphate buffer pH 2.5 to supernatant (1:1 ratio) Centrifuge at 8300× *g*, 10 min, 4 °C, Beckman Coulter Microfuge 18 Microcentrifuge Isolate supernatant and pellet (pellet used for Method 12)
**12.**	Use pellet from Method 11, step e Wash twice with phosphate buffer, pH 2.5 Suspend in phosphate buffer, pH 7.5 (approx. 1.6× the starting volume)

## Data Availability

TLB and clinical/demographic data are available as Supplementary Materials.

## Supplementary Materials

The following supporting information can be downloaded at https://www.mdpi.com/article/10.3390/cancers16244220/s1: Figure S1: Schematic representation of different saliva processing methods; Figure S2: A subset of samples run on the Nano DSC Autosampler System (solid line) and the N-DSC II instrument (dashed line) showing data consistency between instruments; Figure S3: Results of SDS-PAGE analysis of selected saliva samples; Figure S4: Plot of selected PC loadings at each temperature obtained for saliva TLB profiles; Figure S5: Individual TLB profiles of saliva samples obtained from healthy volunteers (Controls) and HNC patients separated into groups based on the overall cancer stage; Figure S6: Individual TLB profiles of saliva samples obtained from healthy volunteers (Controls) and HNC patients separated into groups based on the cancer location; Figure S7: Comparison of TLB profiles of saliva samples obtained from healthy volunteers (Controls) and HNC patients separated into groups based on the cancer T-stage; Figure S8: Comparison of TLB profiles of saliva samples obtained from healthy volunteers (Controls) and HNC patients separated into two groups based on the cancer location: Tonsil and Non-Tonsil, with the Non-tonsil group consisting of samples from patients with FOM, Tongue, and Other locations; Figure S9: Plot of selected PC loadings at each temperature obtained for plasma TLB profiles; Figure S10: Individual TLB profiles of plasma samples obtained from HNC patients separated into groups based on the overall stage of cancer; Figure S11: Comparison of TLB profiles of plasma samples obtained from HNC patients separated into groups based on the cancer T-stage; Figure S12: Individual TLB profiles of plasma samples obtained from HNC patients separated into groups based on the cancer location; Figure S13: Comparison of TLB profiles of plasma samples obtained from HNC patients separated into two groups based on the cancer location: Tonsil and Non-Tonsil, with the Non-tonsil group consisting of samples from patients with FOM, Tongue, and Other locations; Table S1: TLB data and clinical/demographic information for all patient samples included in the study.
