# Supplementary material for: Evaluation of Thermal Liquid Biopsy Analysis of Saliva and Blood Plasma Specimens as a Novel Diagnostic Modality in Head and Neck Cancer
Source: Cancers (Basel). 2024 Dec 18;16(24):4220. doi: 10.3390/cancers16244220 (PMC11674294; doi:10.3390/cancers16244220)
Supplement: Supplementary file 1 [file cancers-16-04220-s001.zip › TLB in ENT - Cancers - Supplementary Figures proof corrected.pdf]

G. Schneider, A. Kaliappan, N. Joos, L. M. Dooley, B. S. Shumway, J. B. Chaires, W. Zacharias, J. M. Bumpous, N. C. Garbett. *Evaluation of thermal liquid biopsy analysis of saliva and blood plasma specimens as a novel diagnostic modality in head and neck cancer*.

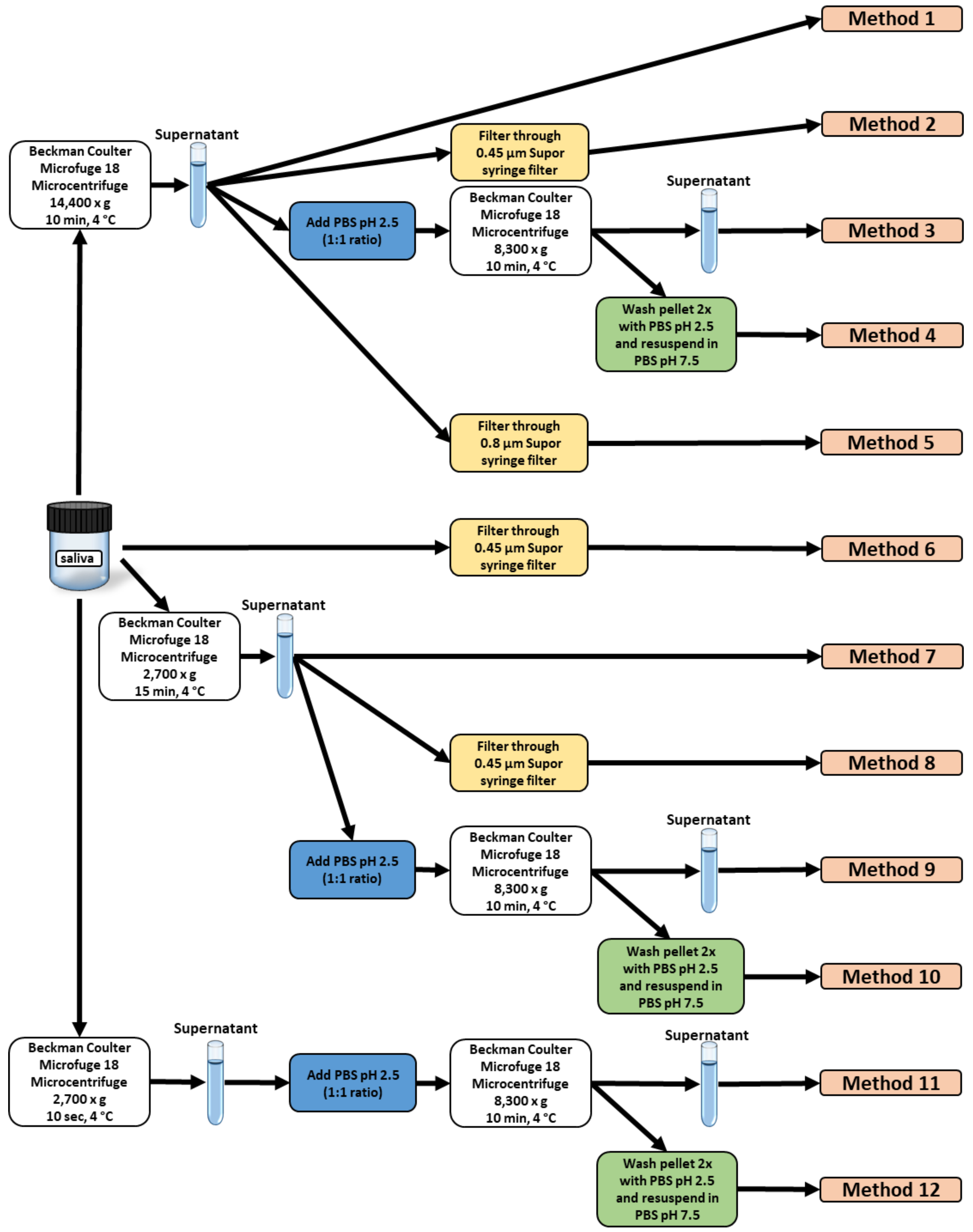

Figure S1. Schematic representation of different saliva processing methods.

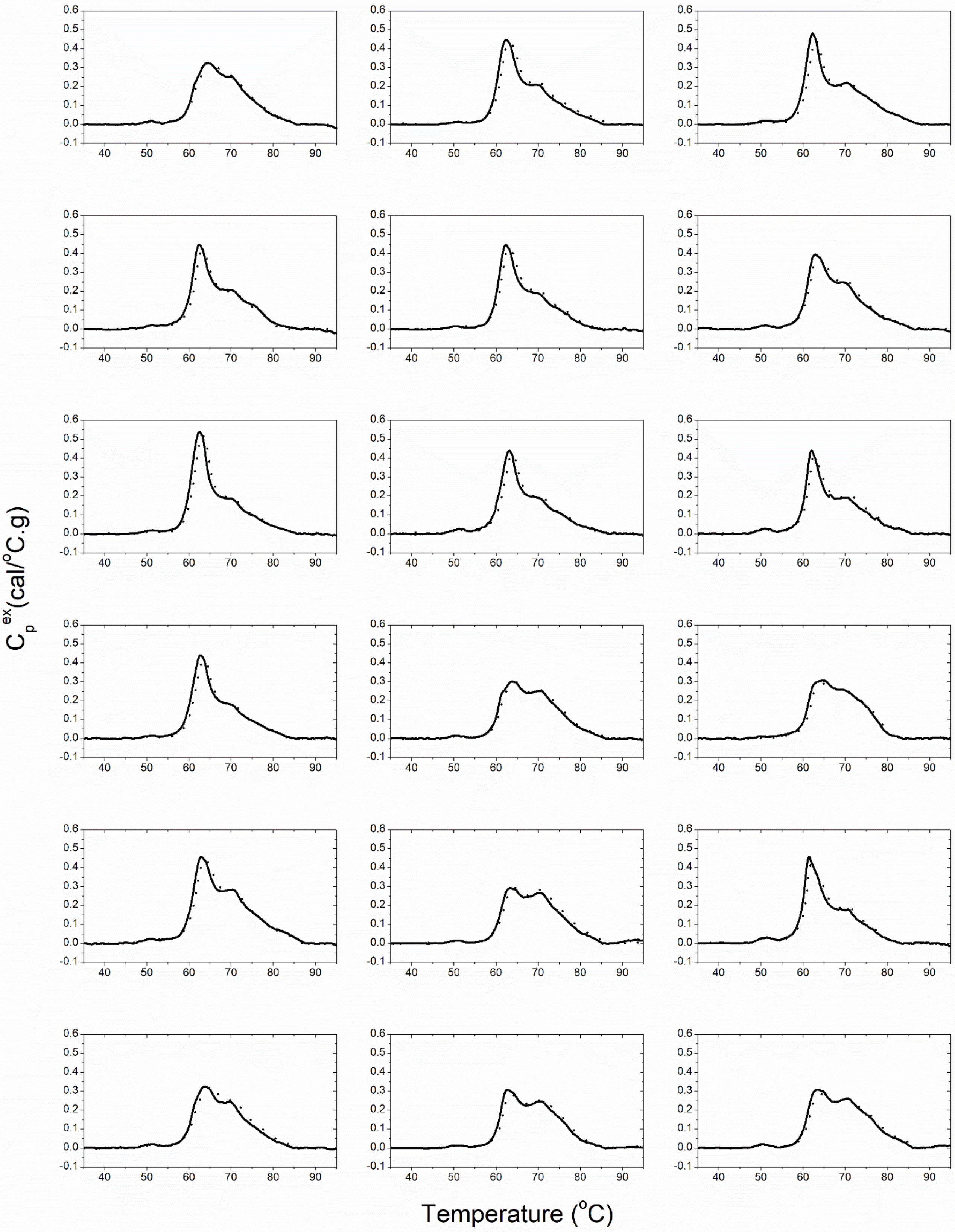

Figure S2. A subset of samples run on the Nano DSC Autosampler System (solid line) and the N-DSC II instrument (dashed line) showing data consistency between instruments.

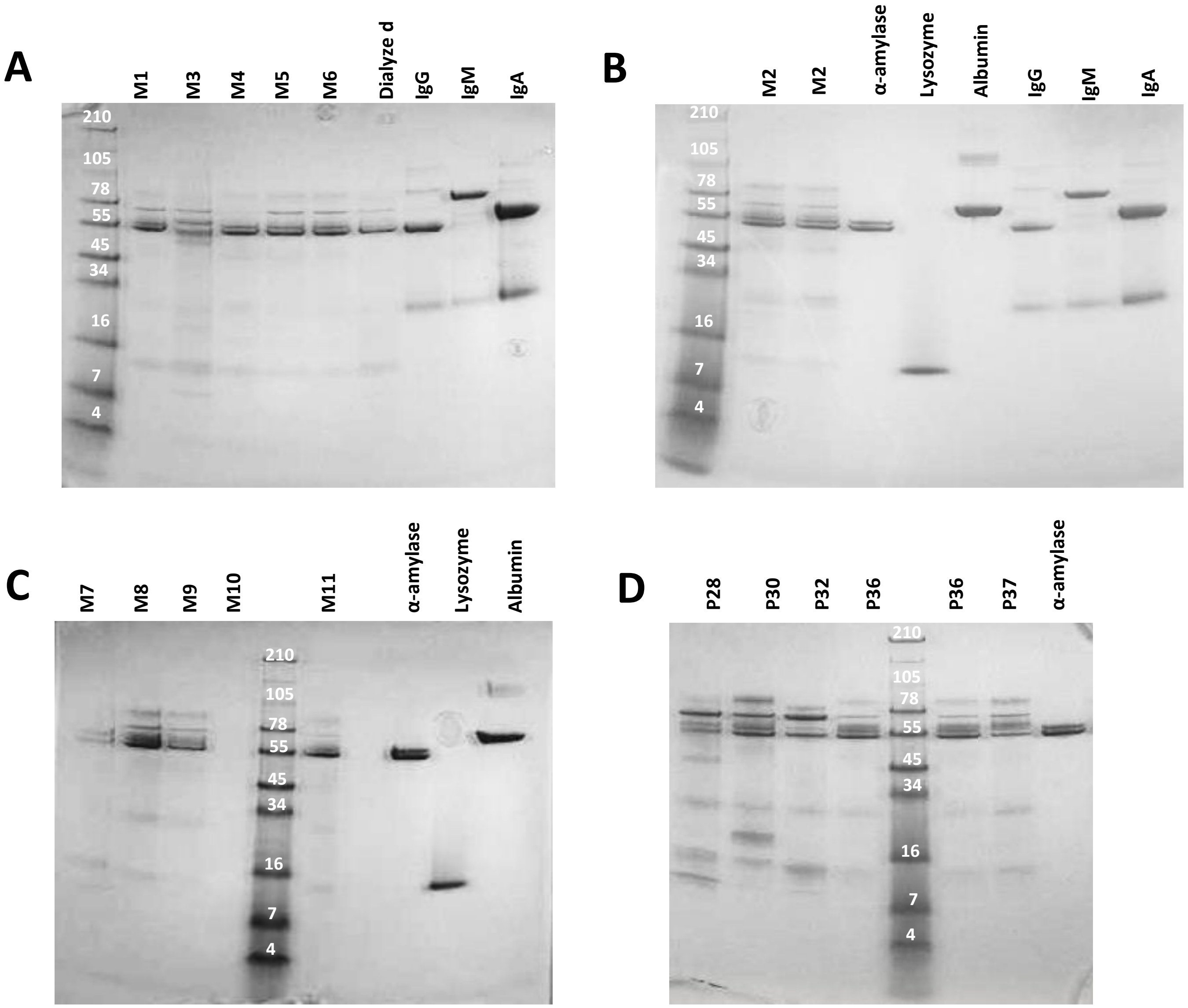

Figure S3. Results of SDS-PAGE analysis of selected saliva samples. Stained gels showing (**A-C**) an unprocessed (but dialyzed) control sample (marked as Dialyzed) compared to the same sample processed using different methods evaluated in this paper, Methods 1-11 (marked as M1-M11); (**D**) selected patient samples (marked with the prefix P) processed using the chosen method (Method 2). Individual purified protein samples representing the most abundant salivary proteins (α-amylase, lysozyme, albumin, IgG, IgA, IgM) were included as reference.

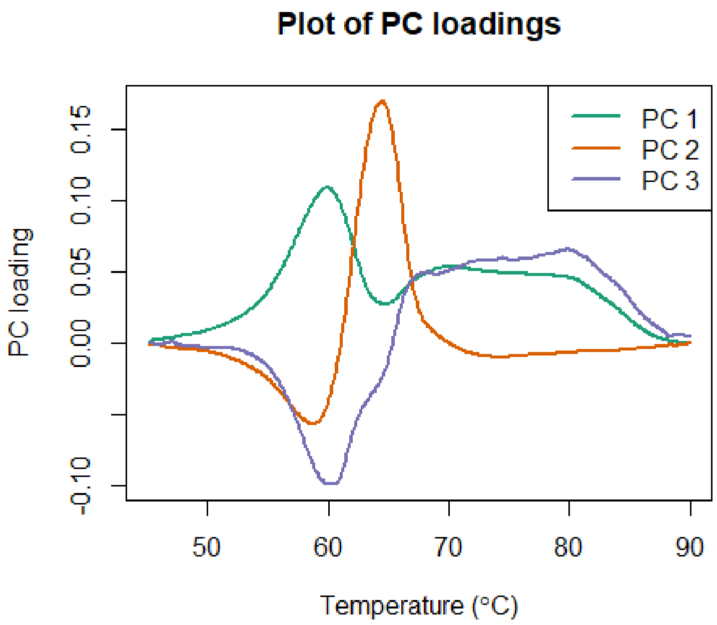

Figure S4. Plot of selected PC loadings at each temperature obtained for saliva TLB profiles.

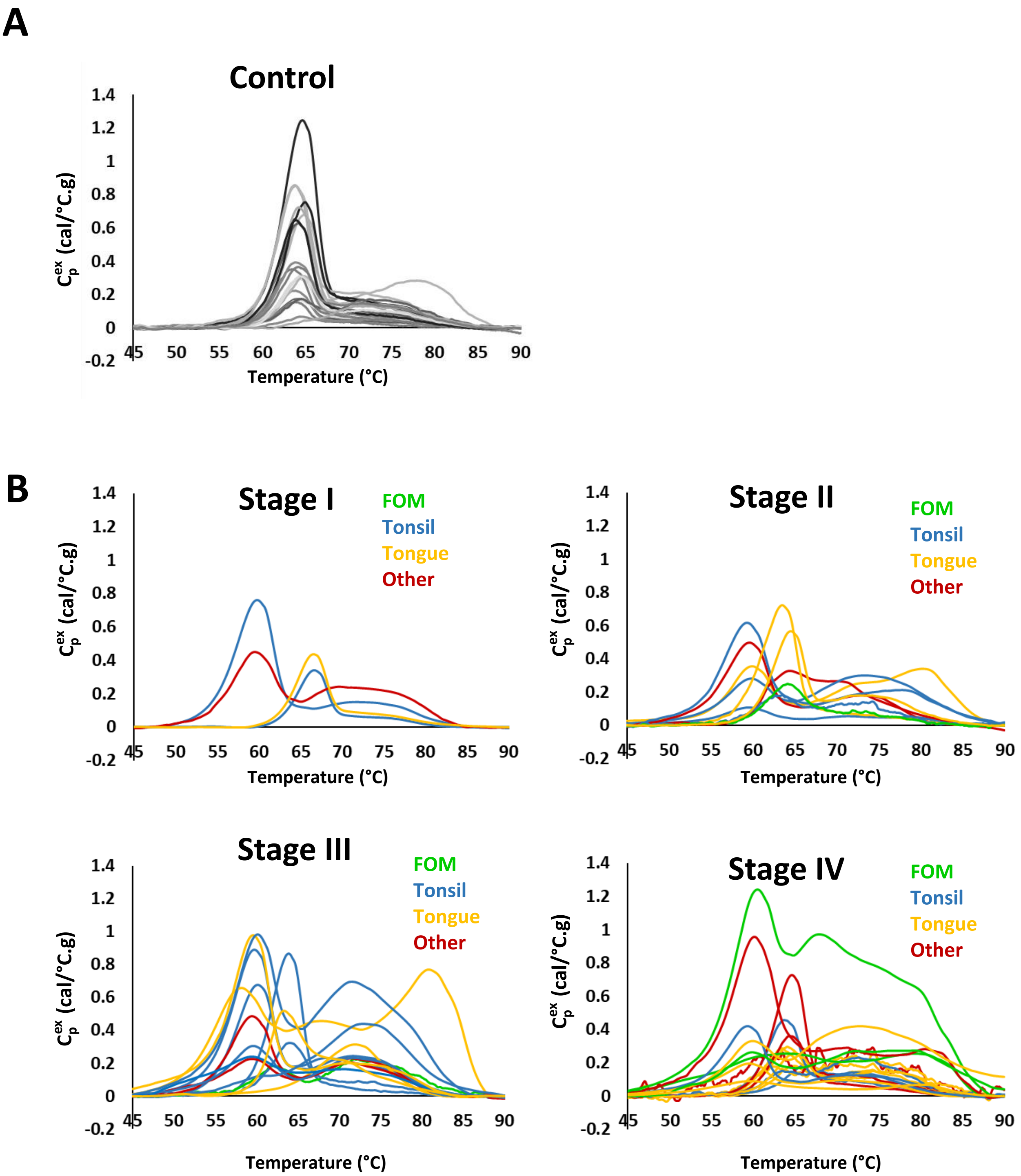

Figure S5. Individual TLB profiles of saliva samples obtained from healthy volunteers (Controls) and HNC patients separated into groups based on the overall cancer stage. (**A**) TLB profiles of saliva samples obtained from Controls. (**B**) TLB profiles of saliva samples obtained from HNC patients separated by the overall stage of cancer.

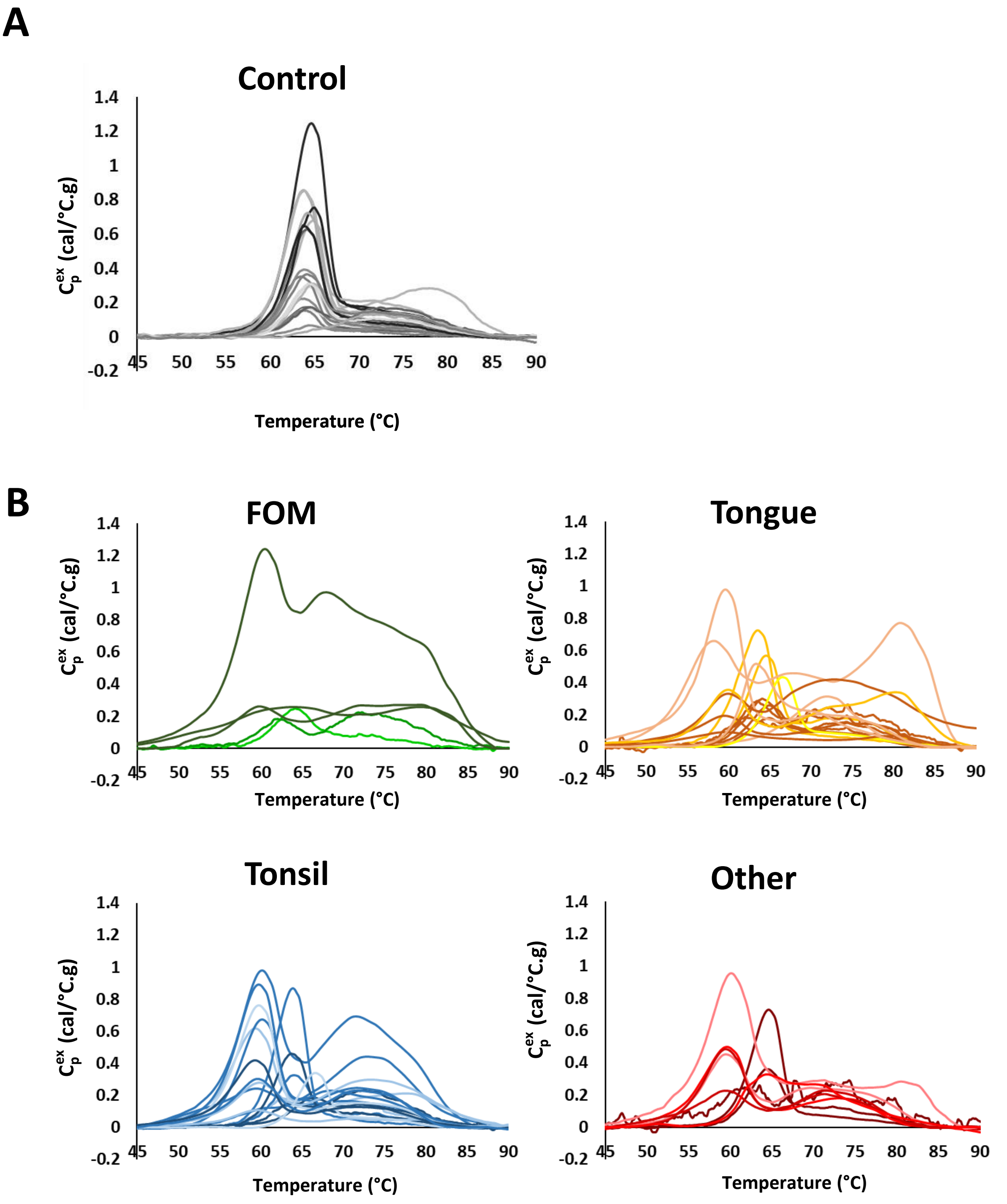

Figure S6. Individual TLB profiles of saliva samples obtained from healthy volunteers (Controls) and HNC patients separated into groups based on the cancer location. (**A**) TLB profiles of saliva samples obtained from Controls. (**B**) TLB profiles of salvia samples obtained from HNC patients separated by the cancer location. The intensity of the color indicates the overall cancer stage, from stage I (lightest) to stage IV (darkest).

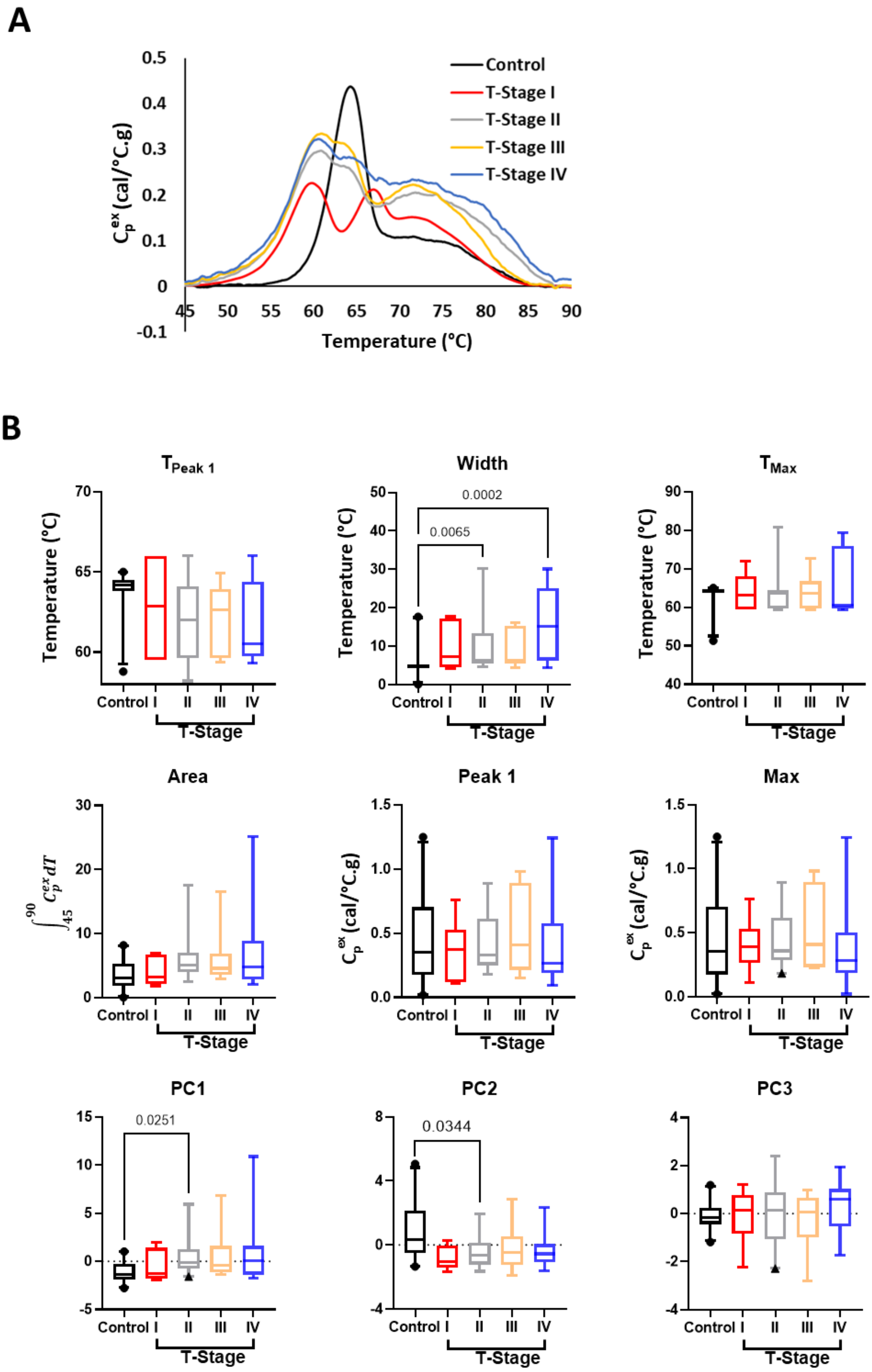

Figure S7. Comparison of TLB profiles of saliva samples obtained from healthy volunteers (Controls) and HNC patients separated into groups based on the cancer T-stage. (**A**) Plot of the mean TLB profile value at each temperature for Controls (n = 21) and patients with different T-stages of HNC (T-stage I, n = 6; II, n = 19; III, n = 10; IV, n = 13). (**B**) Boxplots of metrics and PCs calculated from TLB profiles for Controls and patients with different T-stages of HNC. Unadjusted p-values <0.05 are shown on the graphs.

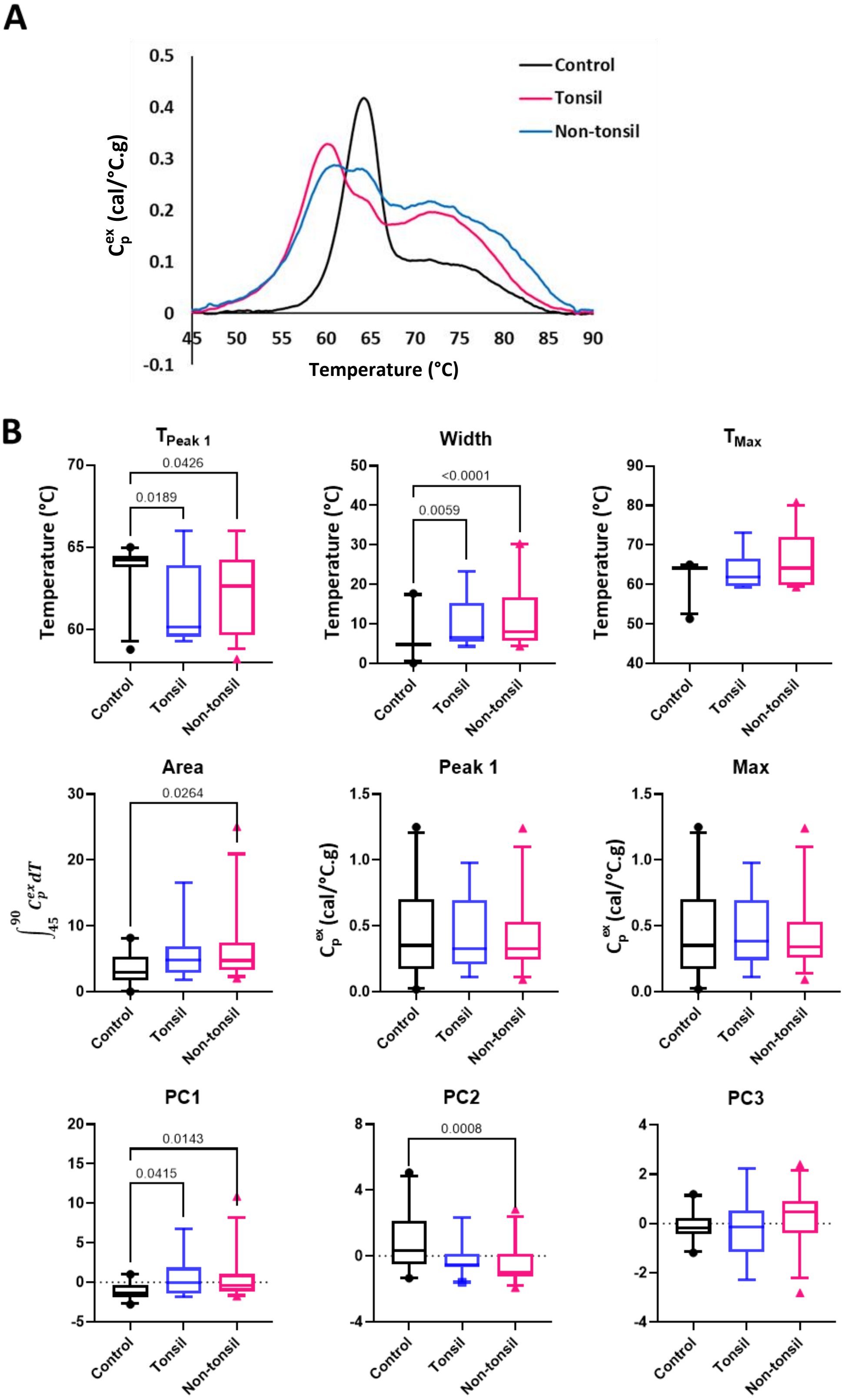

Figure S8. Comparison of TLB profiles of saliva samples obtained from healthy volunteers (Controls) and HNC patients separated into two groups based on the cancer location: Tonsil and Non-Tonsil, with the Non-tonsil group consisting of samples from patients with FOM, Tongue and Other locations. (A) Plot of the mean TLB profile value at each temperature for Controls (n = 21) and patients with different locations of HNC (Tonsil, n = 18; Non-tonsil, n = 30). (B) Boxplots of metrics and PCs calculated from TLB profiles for Controls and patients with different locations of HNC. Unadjusted p-values <0.05 are shown on the graphs.

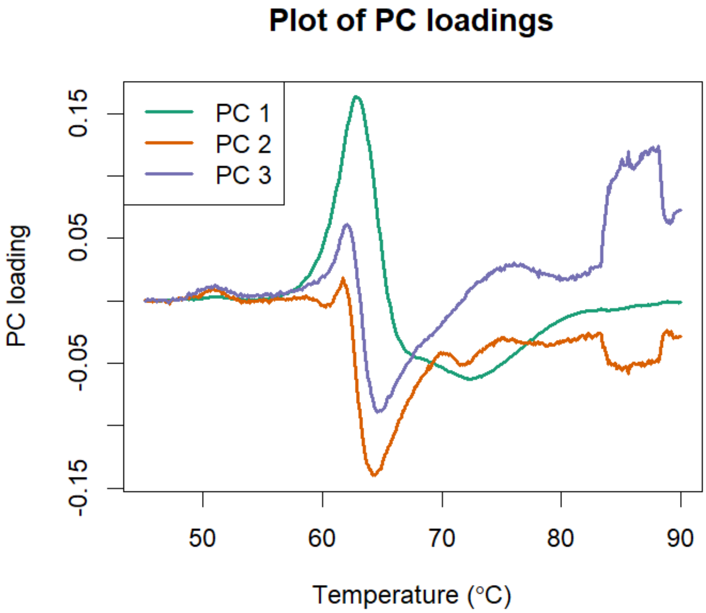

Figure S9. Plot of selected PC loadings at each temperature obtained for plasma TLB profiles.

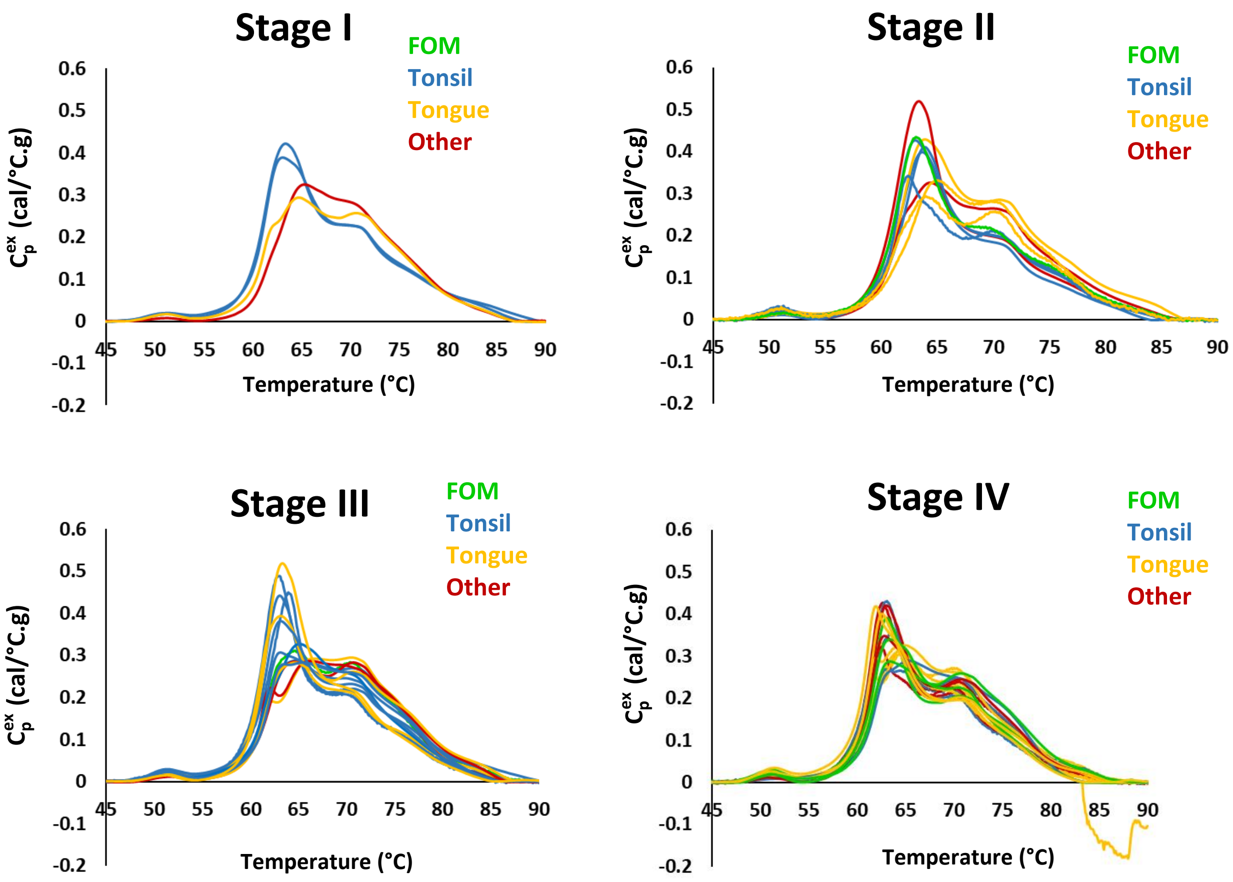

Figure S10. Individual TLB profiles of plasma samples obtained from HNC patients separated into groups based on the overall stage of cancer.

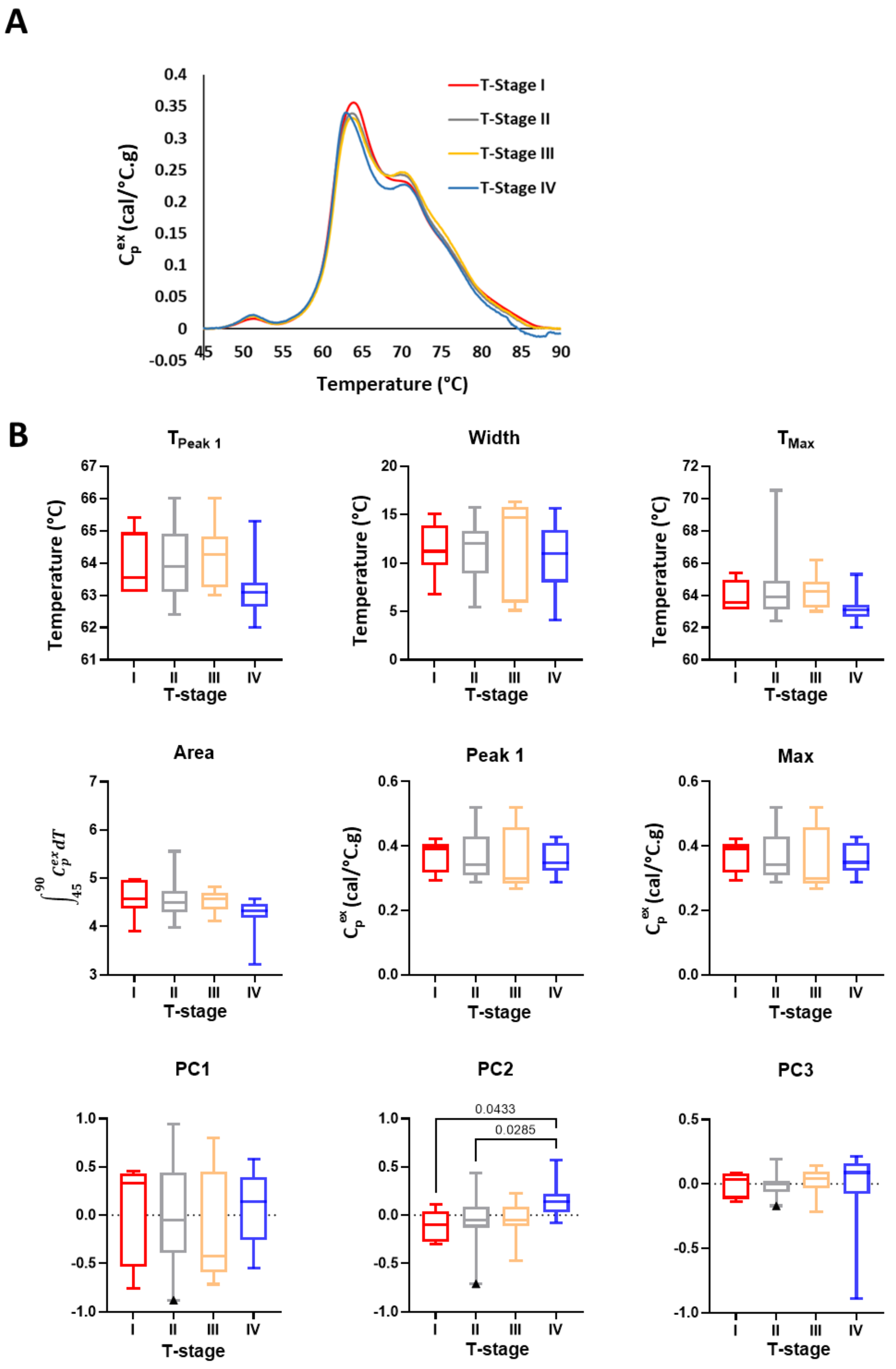

Figure S11. Comparison of TLB profiles of plasma samples obtained from HNC patients separated into groups based on the cancer T-stage. **(A)** Plot of the mean TLB profile value at each temperature for patients with different T-stages of HNC (T-stage I, n = 6; II, n = 19; III, n = 10; IV, n = 13). (**B**) Boxplots of metrics and PCs calculated from TLB profiles for patients with different T-stages of HNC. Unadjusted p-values <0.05 are shown on the graphs.

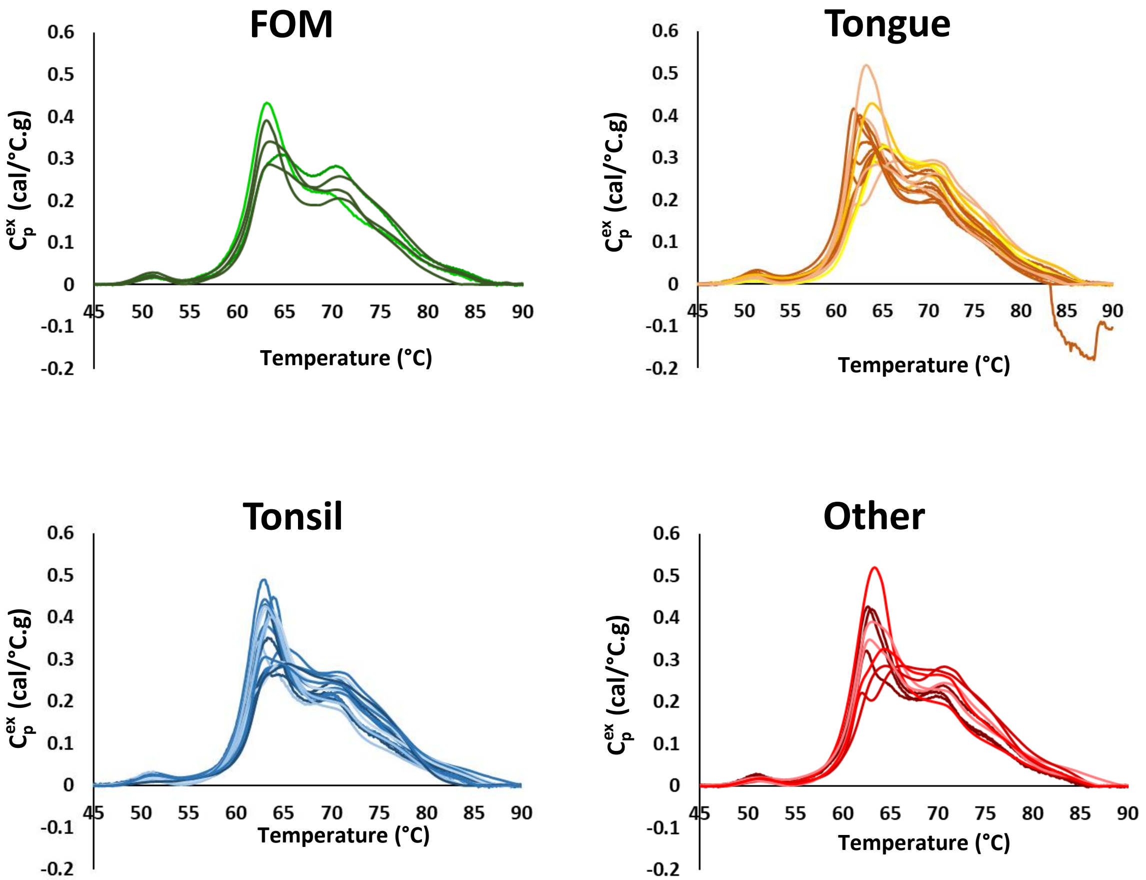

Figure S12. Individual TLB profiles of plasma samples obtained from HNC patients separated into groups based on the cancer location.

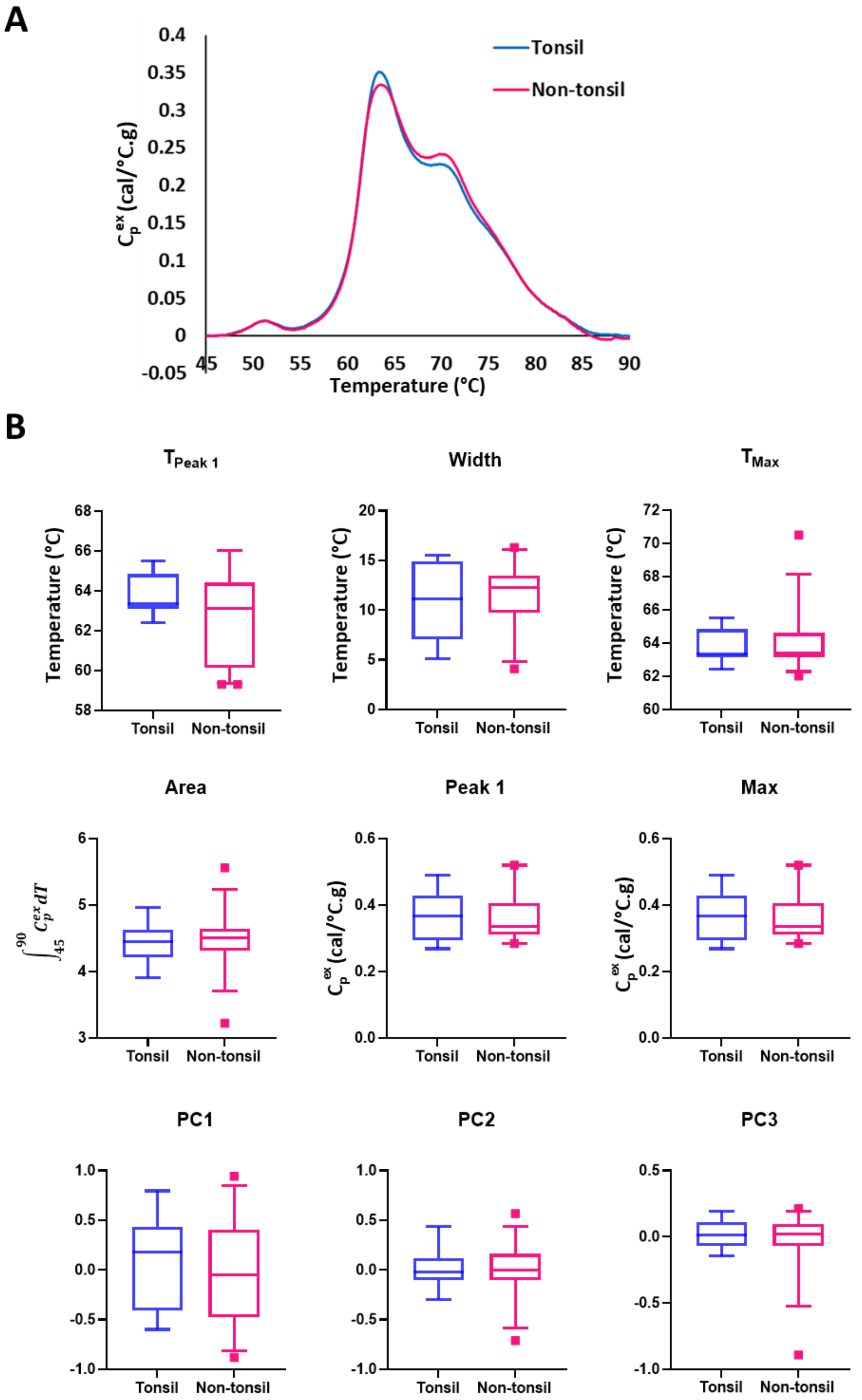

Figure S13. Comparison of TLB profiles of plasma samples obtained from HNC patients separated into two groups based on the cancer location: Tonsil and Non-Tonsil, with the Non-tonsil group consisting of samples from patients with FOM, Tongue and Other locations. **(A)** Plot of the mean TLB profile value at each temperature for patients with different locations of HNC (Tonsil, n = 18; Non-tonsil, n = 30). (**B**) Boxplots of metrics and PCs calculated from TLB profiles for patients with different locations of HNC. No significant differences in metrics or PCs were observed.
